# Nature-Inspired Intelligent Computing: A Comprehensive Survey

**DOI:** 10.34133/research.0442

**Published:** 2024-08-16

**Authors:** Licheng Jiao, Jiaxuan Zhao, Chao Wang, Xu Liu, Fang Liu, Lingling Li, Ronghua Shang, Yangyang Li, Wenping Ma, Shuyuan Yang

**Affiliations:** School of Artificial Intelligence, Xidian University, Xi’an, China.

## Abstract

Nature, with its numerous surprising rules, serves as a rich source of creativity for the development of artificial intelligence, inspiring researchers to create several nature-inspired intelligent computing paradigms based on natural mechanisms. Over the past decades, these paradigms have revealed effective and flexible solutions to practical and complex problems. This paper summarizes the natural mechanisms of diverse advanced nature-inspired intelligent computing paradigms, which provide valuable lessons for building general-purpose machines capable of adapting to the environment autonomously. According to the natural mechanisms, we classify nature-inspired intelligent computing paradigms into 4 types: evolutionary-based, biological-based, social-cultural-based, and science-based. Moreover, this paper also illustrates the interrelationship between these paradigms and natural mechanisms, as well as their real-world applications, offering a comprehensive algorithmic foundation for mitigating unreasonable metaphors. Finally, based on the detailed analysis of natural mechanisms, the challenges of current nature-inspired paradigms and promising future research directions are presented.

## Introduction

For centuries, philosophers and scientists have attempted to create increasingly complex artificial systems by observing, understanding, explaining, and imitating many interesting phenomena in nature. Over hundreds of millions of years, a vast number of species have been born in the universe, the underlying complexity of which is far beyond human comprehension. These species often confront each other with constraints and form part of natural systems. The evolution of complexity in nature tends to follow a low-to-high distribution. So far, natural phenomena, known or unknown, have become a source of creativity in various new disciplines and technological fields [1–5].

With the invention of computers, researchers have consistently looked to nature for inspiration to build general-purpose machines that rivaled or even surpassed humans. Due to its parallelism, derivative-free, and easy scalability, nature-inspired intelligent computing paradigms have proven to be very successful in solving practically complex NP-hard problems and black-box optimization problems such as aerodynamic optimization with an expensive evaluation process [2]. A series of studies [3–5] have shown that these paradigms are powerful methods for cognition and optimization, such as optimizing complex dynamical systems and building cognitive models.

**Fig. 1.**
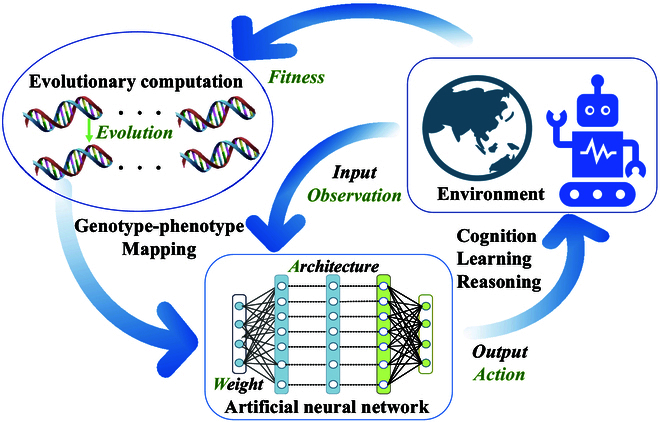
The nature-inspired intelligent computing paradigm provides an approach to evolving the architecture and parameters of an ANN, allowing it to dynamically interact with its environment for cognition, learning, and reasoning.

Nature-inspired intelligent computing paradigms model various phenomena in the natural world to solve various practical problems faced by humans, effectively building a “bridge” between natural disciplines and artificial machines. For example, in nature, the evolved human brain allows humans to participate in complex social collaborations. Similarly, it is a hot application for the nature-inspired intelligent computing paradigm to use evolutionary algorithms (EAs) to optimize brain-inspired artificial neural networks (ANNs) so that disorganized neural networks can evolve into general-purpose neural networks [6–8]. As shown in Fig. 1, ANNs can be employed for cognition, learning, and reasoning by evolving the architecture *A* and parameters *W* of the ANNs.

In general, nature-inspired intelligent computing paradigms have characteristics that nature possesses, such as randomness, nonlinearity, ergodicity, self-organization, adaptability, diversity, stability, and high parallelism. Due to the improvement in computing and information processing capabilities of computers over the past decade, nature-inspired intelligent computing paradigms are employed to solve various complex problems in the real world. For instance, material design can be optimized using genetic algorithms (GAs), while material design can be effectively addressed through GA methods [9]. River flow forecasting has been successfully performed using EAs and particle swarm optimization (PSO) techniques [10]. Additionally, quantum-inspired gray wolf optimization (GWO) has been applied for breast cancer diagnosis [11], and intelligent inspection robots for obstacle avoidance have been constructed using improved PSO [12]. Although providing systematic solutions for highly complex problems, nature-inspired intelligent computing paradigms are dwarfed by the diversity, stability, and adaptability of nature.

**Fig. 2.**
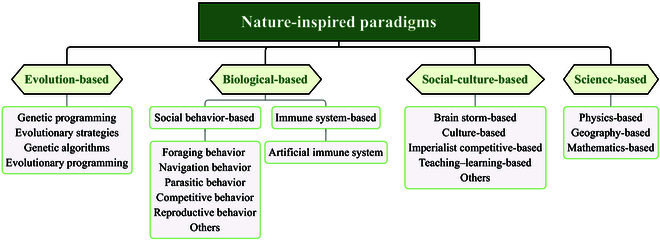
Nature-inspired intelligent computing paradigms are divided into 4 categories: evolution-based, biological-based, social-cultural-based, and science-based.

Although existing reviews [13–26] have offered valuable insights, a comprehensive survey that systematically encompasses all categories within the nature-inspired intelligent computing paradigm is still lacking. Furthermore, the connections between natural mechanisms and these paradigms, as well as the deep mathematical or physical principles underlying them, are not fully explored. This paper aims to fill the gap by presenting a wide-ranging exploration of nature-inspired intelligent computing research, including evolution-based, biological-based [encompassing social behaviors and immune systems (ISs)], culture-based, and science-based methods as shown in Fig. 2. We delve into various nature-inspired intelligent computing paradigms from a natural mechanism perspective, focusing on the origins of the algorithms. This perspective reveals the nuances that distinguish natural mechanisms from nature-inspired intelligent computing paradigms, uncovers commonalities, and encapsulates key design principles, updating procedures, and typical applications. In addition, this paper summarizes potential future challenges and discusses prospective advancements in several application areas. The main contributions of this paper are as follows:

1. Comprehensive overview: We provide a comprehensive overview of the natural mechanisms of the nature-inspired intelligent computing paradigms and divide them into 4 major categories: evolution-based, biological-based, social-cultural-based, and science-based. This holistic approach provides a broader understanding of the field.

2. Characteristics and real-world applications: This paper investigates the commonalities as well as classic real-world application examples of nature-inspired intelligent computing paradigms. These reviews reveal connections between natural mechanisms and nature-inspired intelligent computing paradigms.

3. Connections between natural mechanisms and paradigms: We delve into the connections between natural mechanisms and the corresponding computational paradigms, uncovering the fundamental principles and design methodologies of algorithms inspired by nature. This perspective highlights the nuances that distinguish natural mechanisms from nature-inspired computing paradigms and encapsulates key design principles, updating procedures, and typical applications.

4. Challenges and future directions: The current challenges and future potential research directions based on the nature-inspired intelligent computing paradigm are presented. This comprehensive discussion provides valuable future exploration for general artificial intelligence (AI) technology.

The organization of this paper is as follows. In the “Related Work” section, we review related work on nature-inspired intelligent computing paradigms. The “Evolution-Based Paradigms” section presents the natural mechanisms of evolution-based paradigms, while the “Biological-Based Paradigms” section discusses those of biological-based paradigms. The fundamental natural mechanisms of social-cultural-based paradigms are detailed in the “Social-Cultural-Based Paradigms” section, and the natural mechanisms of science-based paradigms are outlined in the ”Science-Based Paradigms” section. The “Challenges and Potential Future Research Directions” section points out the challenges of the nature-inspired intelligent computing paradigms and potential future research directions. Finally, the “Summary and Outlook” section concludes this paper.

## Related Work

Many surveys and reviews have been published to highlight the pivotal role of nature-inspired intelligent computing paradigms, each of which provides valuable insights and a comprehensive overview of the field. These scholarly contributions enrich our understanding of the fundamental concepts, recent advancements, and prospective future trajectories in these paradigms.

In the field of nature-inspired intelligent computation, researchers have delved into a myriad of paradigms, with their own unique perspectives. For example, Kumar and Singh [15] and Sachan and Kushwaha [16] offered comprehensive reviews covering evolution, swarm intelligence, biology, and scientific approaches, and scientific viewpoints. Tang et al*.* [17] delved specifically into swarm intelligence algorithms, whereas Chelly Dagdia et al*.* [14] focused on the interplay between biology and computer science. Many works [13,19] emphasized the connection between evolutionary methodologies and evolutionary computation. Torres-Treviño [18] explored biological and viral standpoints. Omidvar et al*.* [4,27] summarized metaheuristics for large-scale problems. Recently, Kudela [28] analyzed the classic evolutionary paradigms on benchmark functions and gave several suggestions for improvement. Gharehchopogh [29] provided a comprehensive summary of quantum-inspired metaheuristics and their applications in science and engineering. For paradigm descriptions, the existing surveys focused on additional procedural steps, variations of algorithms, and their diverse applications [15–18]. Alternatively, some work [13,14,19] opted for a more synthesized overview of specific algorithm classes. These explorations highlighted the powerful applicability of nature-inspired intelligent computing paradigms, illustrating its expansive scope and dynamic evolution.

While existing reviews primarily concentrated on the evolution of nature-inspired intelligent computing paradigms [13,14,16,17,19], many studies [24,25] have shifted the discourse toward the open challenges that require attention in this field, such as the risk of unreasonable “metaphors” [30–33]. Additionally, there has been a notable surge in publications dedicated to the application of nature-inspired intelligent computing paradigms across diverse domains. These studies encompass neural networks [13,34], reinforcement learning (RL) [2], unbalanced classification [35], feature selection [36], image processing [37], image segmentation within biomedical contexts [20–22], and applications in the process industry [23]. These works indicated a broadening interest and applicability of nature-inspired intelligent computing paradigms, underscoring their substantial in emerging technological fields.

This survey builds upon the valuable insights from existing reviews by offering a more comprehensive and integrative perspective on nature-inspired intelligent computing paradigms. It distinguishes itself by systematically encompassing all major categories of nature-inspired computing paradigms, including evolution-based, biological-based, social-cultural-based, and science-based methods. The work delves into the deep connections between natural mechanisms and their corresponding computational paradigms, uncovering fundamental principles and design methodologies. Beyond surface descriptions, it explores the deep mathematical principles and classic real-world application examples, providing a deeper understanding of their origins, operational mechanisms, and practical applications. Additionally, the survey offers a detailed discussion of current challenges and future research directions, presenting a comprehensive roadmap for future exploration and potential advancements in the field. By focusing on these aspects, the survey aims to provide a holistic and integrative understanding of nature-inspired intelligent computing paradigms, bridging gaps in the existing literature and paving the way for future research.

## Evolution-Based Paradigms

Evolution provides a creative source of power for complex and delicate problems. Inspired by the origin of species proposed by Darwin [38], evolution-based paradigms have become one of the best-known concepts in the field of nature-inspired intelligent computing paradigms [39]. In a sense, evolution is independent of various physical media and occurs in the development of anything. Like biological evolution, evolution-based paradigms exhibit surprising experimental results on complex problems [40,41]. In this section, the mechanisms of natural evolution and artificial evolution paradigms, as well as the gap between them are systematically reviewed.

### The natural mechanism of evolution-based paradigms

Since the Big Bang, all kinds of primitive life have gradually reproduced and evolved into more complex life forms on land, sea, and air. It can be observed that evolutionary adaptation leads to a diversity of organisms. Over time, species make adaptive adjustments to environmental changes. Differences in genes cause individuals of the same species to exhibit different variation characteristics. Individuals who successfully adapt to their environment are more likely to survive and reproduce so that the good genes are passed on to their offspring. Those individuals who cannot adapt to the environment are eliminated. Inspired by this evolutionary process, the powerful creativity of evolution is used to solve complex problems in many fields such as natural cognition, scientific exploration, and social development. For nearly a hundred years, several underlying mechanisms of inheritance have been revealed by quantitatively exploring the spread of variant alleles, leading to a unified interpretation of biodiversity on Earth [13,14]. Key drivers of evolution include genotype-to-phenotype mapping, mutation, genetic drift, recombination, gene flow, and natural selection.

Genotype-to-phenotype mapping is fundamental to the evolutionary process of micronized analysis. Gene refers to the basic material unit that carries genetic information. The properties of an organism are inherited through genes or other mechanisms. Humans contain about 20,000 to 25,000 genes. The interaction of genotype and environment can produce a set of observable traits, called phenotypes, such as biological structure or behavior. By exploring the relationship between genotype and phenotype, the functions of many genes have been revealed [42]. Mapping between genotypes and phenotypes is a large-scale complex nonlinear problem, which is difficult due to too little data on genotypes, insufficient description of phenotypes, and the underlying complexity of regulating cellular functional networks [43]. In general, genotype and phenotype are not in a one-to-one mapping and they are both regulated by gene regulatory networks and phenotypic genetic traits [44]. Most traits are controlled by multiple interacting genes [45]. For example, the combinatorial complexity of potential epistasis linking in proteins makes relating genotype and phenotype difficult [46]. In addition, the organism itself also possesses phenotypic plasticity [47], that is, when the genotype remains unchanged, the phenotype may also change and be passed on to the next generation.

Mutation, genetic drift, recombination, and gene flow are the creative sources of evolution, providing the raw material for natural selection. In the process of reproduction, mutation is the change of genetic material at a certain frequency, such as point mutation caused by a single base [48] and deletion, insertion, and rearrangement of multiple bases [49]. The triggers include replication errors during cell division [49], chemicals (nitrous acid [50], aflatoxin [51], etc.), radiation (x-rays [52], ultraviolet light [53], etc.), or the effects of viruses [54]. Mutation frequencies vary widely across species, strongly influenced by the environment and the genetic information of the organism [55]. Exploring mutation frequencies can prioritize genes and pathways in a manner that increases public health benefits [56].

Mutations are universal, random, rare, reversible, nondirectional, independent, and recurring. The effects of mutations on an individual may be beneficial [57], detrimental [58], or both [59]. Beneficial mutations increase the fitness of an organism. In recent studies, gene editing techniques could allow the introduction of beneficial mutations to improve genetic diseases [57], which further reveals the mechanism of complex gene expression, while harmful mutations can greatly reduce the fitness of organisms, for example, mutation-induced genes may lead to various diseases [58]. Mutations that are neither beneficial nor harmful to the individual are called neutral mutations. In the absence of natural selection, neutral mutations still occur with a certain frequency and then accumulate gradually between species. Although neutral mutations have no direct effect on phenotype, studies [60] have shown that a robust neutral mutation may be more likely than a vulnerable mutation to spread in a large and diverse population and the neutral mutation sequence follows the “random walk with maximum entropy”.

Genetic drift refers to the random variation of allele frequencies in a population between each generation. Even in the absence of selection, genetic drift causes different populations of the same genetic structure to evolve into new populations with different sets of alleles [61]. Recombination is the exchange of genetic material between organisms, which allows offspring to own different characteristics from the parent. In general, prokaryotes can directly exchange genes with each other through conjugation, while eukaryotes perform gene recombination by exchanging chromosomes during meiosis and mitosis. The phenomenon of recombination is used for DNA repair to maintain genome integrity [62]. Gene flow is the exchange of genes that occurs between populations or species, which is a new source of variation in populations or species. The transfer of genetic material between species leads to the formation of new species. For example, the eukaryotic genome is a fusion of different prokaryotic genomes [63].

**Fig. 3.**
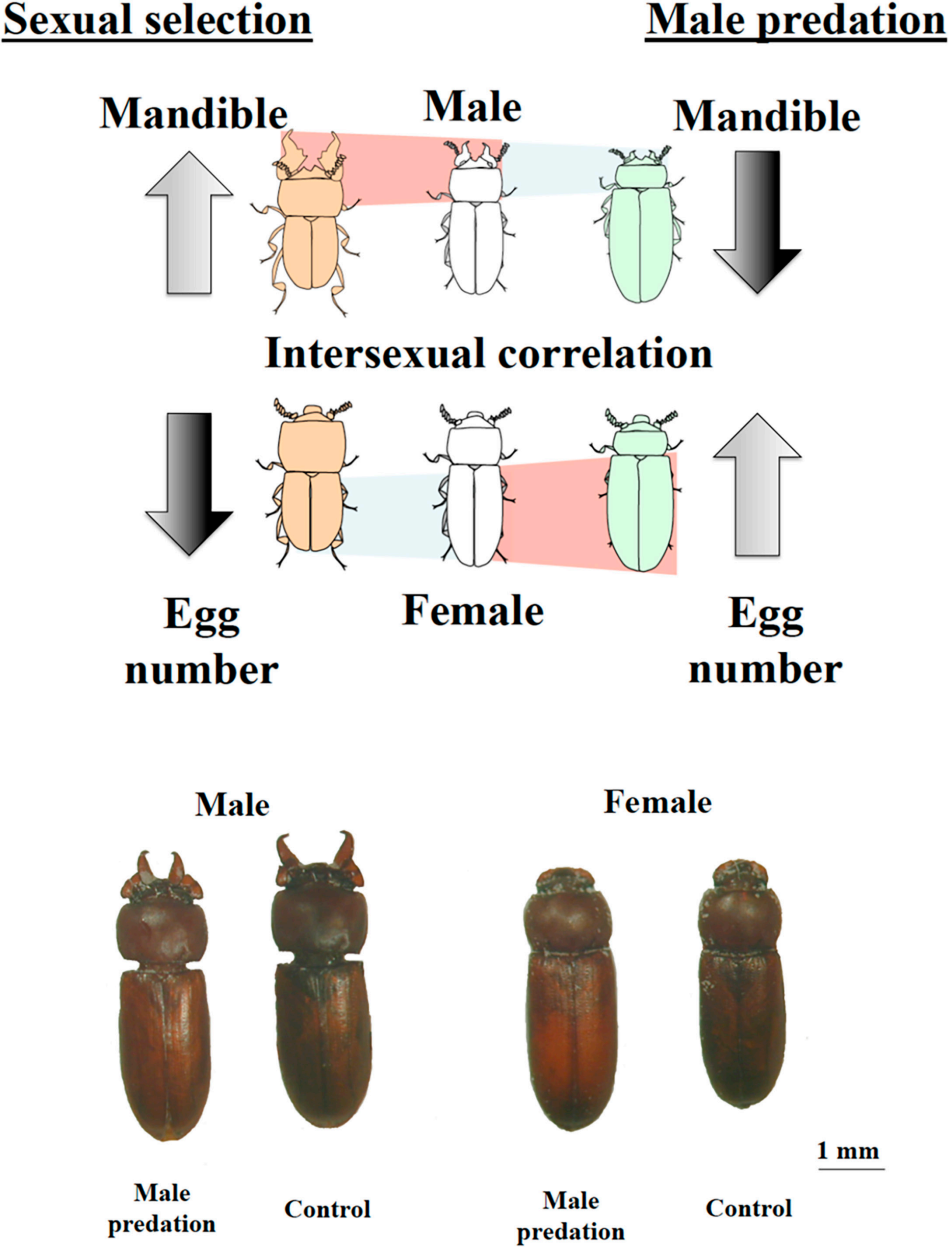
Male and female phenotypes result from male-limited predation and male sexual selection. Sexual selection leads to enlarged mandibles in males, and male-restricted predation leads to larger abdomens in both males and females (image from [65]).

Natural selection is the core force of evolution. Through natural selection, those traits with higher survivability become more prevalent in populations. Fitness is employed to measure an organism’s ability to survive and reproduce. If an organism is more likely to survive and reproduce quickly, it has a higher fitness, and vice versa. Natural selection can act on different hierarchies, such as genes, cells, individuals, biota, and species, separately or simultaneously [64]. Furthermore, sexual selection is a special case of natural selection. Traits that have evolved through sexual selection are particularly prominent in the males of some animal species. As shown in Fig. 3, recent studies [65] have shown that it is even possible to increase female fitness by reversing the exaggeration of male sexual selection traits.

### Evolution-inspired paradigms

The evolution-inspired paradigm is one of the oldest and most well-established branches of natural computation, originally developed for the study of adaptive systems rather than function optimization. Evolution-inspired paradigms encompass a wider variety of systems than function optimizers. The classic evolution-inspired paradigm encompasses 4 domains: GA, evolutionary programming (EP), evolutionary strategies (ES), and genetic programming (GP). In fact, hybrid paradigms of these 4 methods are becoming more and more popular. Their joint development leads to the prosperity of the evolution-inspired paradigm, which has been widely used in various engineering optimization problems [66].

**Fig. 4.**
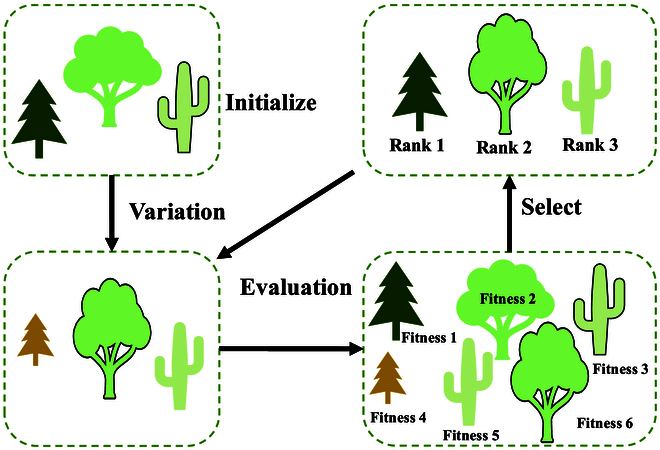
A general framework based on the evolution-inspired paradigm.

Figure 4 presents a general framework based on the evolution-inspired paradigm. Inspired by genetic evolution, the evolution-based paradigm includes initialization, variation, evaluation, and selection. The whole process acts on many individuals or chromosomes. Each individual or chromosome represents a potential solution to a problem and is encoded according to the problem properties. Through reproductive operations (crossover and mutation) between individuals, those new traits or genes are constantly emerging in the population. There are some properties of the parent and new properties in the new individuals. Fitness is employed to measure the individual’s performance on the problem to be solved. Individuals with high fitness gradually dominate the population, while individuals with lower fitness values are eliminated. Over a certain number of generations, individuals evolve to maximize their performance on the problem. Inspired by the concept of evolution, a series of different evolution-based paradigms have been proposed. These methods have different ways of expressing information, which are presented as follows.

Genetic algorithm. GA [67], a classical paradigm inspired by evolution, is originally employed to describe the adaptive process of natural systems [68], and later widely applied in learning and optimization. GAs maintain a population approaching the optimal solution by modeling the selection, crossover, and mutation mechanisms in natural evolution.

Classical GAs employ binary strings to represent an individual. Subsequently, the representation of individuals is also extended to other types, such as integer encoding, real encoding, and sequential encoding. Unlike the complex genotype-to-phenotype mapping in natural evolution, individual representations in GAs tend to be one-to-one.

For the selection process, the algorithm designs uncertain selection operators such as roulette and binary tournaments. The update process of the population is often divided into 2 strategies: generational replacement and steady-state replacement [14]. Generation replacement is the complete replacement of the parent with all the resulting children. Steady-state replacement replaces only a portion of the parent with the child. In survival selection, selection pressure refers to the degree to which excellent individuals are favored. The greater the selection pressure, the faster the population converges and the search space may not be fully explored. As the core driving force of evolution, suitable selective pressure ensures the spread of excellent genes and the elimination of inferior genes, pushing populations toward better solutions [69].

Crossover-dominant and mutation-secondary strategies are employed in GAs. Crossover operators include single-point, multi-point, and uniform crossover. Mutation operators include single-point, multi-point, and uniform mutation. In GAs, various problem-specific genetic operators can be designed to improve the performance of the algorithm. For example, in numerical optimization, simulated binary crossover [70] and polynomial mutation [71] are proposed to explore the search space of real numbers.

In addition, unlike the natural evolution theory, some local search strategies are introduced into the GAs to guide the population to speed up the evolution according to the specific prior knowledge of the solving problem, such as the memetic algorithm [72]. Recently, many works utilizing neural networks to learn and distill GAs have been proposed to improve performance and generalization [73–75].

Evolutionary strategies. ESs were proposed by Rechenberg [76] for solving continuous optimization problems. The individuals in its population are represented by genetic material and control parameters. The control parameters are usually a set of random vectors that are normally distributed. This parameter describes the specific behavior of the individual. In most ESs, the selection process is deterministic based on fitness ranking. In the process of evolution, control parameters and genetic material evolve at the same time.

The covariance matrix adaptation evolution strategy (CMA-ES), one of the most theoretically complete ESs [77], is widely used in various nonlinear or nonconvex continuous optimization problems. In CMA-ES, new individuals are generated from a multivariate normal distribution. Crossover is a process to select a new mean for the distribution, and variation is equivalent to adding a perturbation with zero means. The relationship between variables in distribution is represented by a covariance matrix. The adaptation of the covariance matrix is a method of automatically updating the distribution. On the quadratic function, this adaptive covariance matrix approximates the inverse of the Hessian matrix in the quasi-Newton method. The latest research [78] shows that CMA-ES is an information geometry optimization algorithm (IGOA) whose parameter distribution is a multivariate normal distribution. IGOA transforms any family of smooth parameter probability distributions over the search space into a black-box optimization algorithm in continuous time. The resulting IGOA is a flow of ordinary differential equations that guide the adaptive transformation of the objective function for natural gradient ascent. This idea provides a solid theoretical foundation for the ESs. Recently, Riemannian natural gradients and Hessian approximation have been further extensively studied to improve the theoretical performance of ESs [5,78–81].

Evolutionary programming. EP [82] employs evolution as a learning process to generate a wide range of AI machines. It emphasizes changes in group behavior in natural evolution rather than genotype, so mutation is the only reproductive operator. Like ESs, probability distributions are used to guide the mutation process and distribution parameters are adaptively updated in EP. In EP, the selection mechanism of the parent is deterministic, while the replacement process is uncertain, such as Boltzmann selection and proportional selection. EP currently has no fixed structure, and the boundaries with several other paradigms are increasingly blurred.

Genetic programming. GP [83], a technique for automating the generation and selection of computer programs to accomplish user-defined tasks, is regarded as an important extension of GAs. A nonlinear structure like a tree is used to represent programs in the original GP. Similar to GAs, a group of randomly generated programs gradually evolve into programs adapted to specific tasks by operations such as crossover, mutation, and selection. GPs based on different data structures are also designed to generate modern computer programs, such as Python code generation [84]. With the rapid development of deep learning, automatic deep learning has become an important application fileds of GP [85–87]. The performance of deep learning is heavily influenced by its parameters as well as its architecture. Automatic deep learning is a constrained optimization problem with multi-level multi-objective high-dimensional characteristics, and its inherent nonconvex and black-box properties make GP one of the potential solutions. Recently, large language models (LLMs) have demonstrated extraordinary sequence learning capabilities [88]. Accelerating GP using LLMs has been used to solve various complex mathematical reasoning and engineering optimization problems [89–92]. Due to the flexibility and simplicity of language representation, LLM-assisted GP is expected to address more complex scenarios in open environments.

## Biological-Based Paradigms

**Fig. 5.**
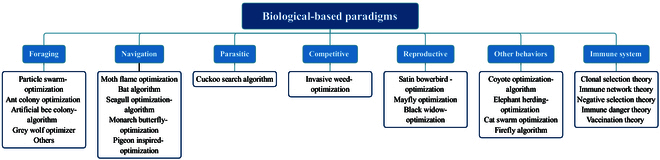
The classification of biological-based paradigms including foraging, navigation, parasitic, competitive, reproductive, immune system, and others.

Biological-based paradigms stem from the study of “emergent” phenomena in nature. Such paradigms usually consist of a group of simple individuals that communicate with each other and their environment [93]. Due to the highly self-organizing, parallel, stochastic, and simple implementation properties, biological-based paradigms have received broad attention among scholars and applied to various optimization problems. As shown in Fig. 5, this section starts from social biomechanics and extends a family of related paradigms based on social behaviors as well as the artificial immune system (AIS).

### Social animal and swarm behavior

The movement of species aggregation is called swarm behavior [94]. All these swarm behaviors are observed in nature, with ants cooperating with each other to forage and build complex burrow passages, birds avoiding collisions in flight to achieve coordination, and bees collecting nectar from food sources (flowers) with great efficiency. The “symphony-like” way in which swarms cooperate has always fascinated researchers. Every swarm consists of a number of simple entities with local interactions (including with the environment). The emergence of complex or mesomorphic behaviors and the ability of swarms to achieve significant results are the product of a combination of simple or microscopic behaviors.

Social learning has been found in a variety of social animals or plants, where species learn by observing the behavior and experiences of other individuals as well as by the transmission of information between individuals within intraspecific species [95]. Some social animals have evolved the flexibility and intelligence to deceive or benefit from other animals, even to predict others’ behaviors [96]. Research [97] showed that wild vervet monkeys are capable of learning what to eat from the more experienced individuals in their social group. Social animals coordinate their behavior in groups, and their nervous systems are likely to do the same [98]. The theoretical foundations of biological research inspired researchers with numerous insights.

### Social behavior-based paradigms

The social behavior-based paradigm models the cooperative relationships of intraspecific individuals. In 1989, Beni and Wang [99] first proposed swarm intelligence in the context of cellular robotic systems and considered swarm intelligence as the process of combining computation and dynamics. Subsequently, swarm intelligence was extended to the collective and social behavior of insects and other animals, bionic “nature” (i.e., physical or biological systems) to build adaptive, decentralized, flexible, and robust artificial systems as a paradigm for problem solving [100].

**Fig. 6.**
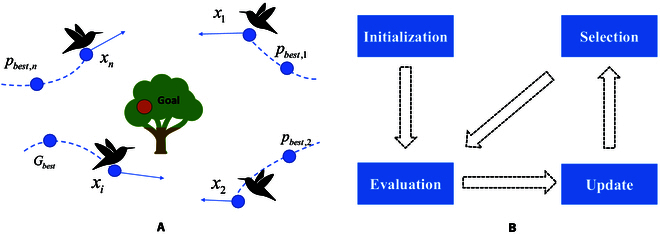
A general framework based on the biological-inspired paradigm. (A) Process of updating in the PSO algorithm, where *P_best_* and *G_best_* represent the local and global optimal positions of the particle swarm *x_i_*, respectively. (B) Main steps of the biological-inspired paradigm.

According to the forms of collective animal or plant behavior, social behavior-based paradigms can be classified as foraging, navigation, parasitism, reproduction, competition, and others. The main characteristics of paradigms include self-organization, indirect communication, high parallel distribution, stability, and adaptability [101]. This family of paradigms is composed of a population of agents capable of interacting with each other or with the environment, where the agents interact cooperatively by using simple local rules in a decision space to accomplish a certain task [14]. Figure 6 presents a general framework based on the social behavior-based paradigm, with the update step of the PSO algorithm as an example. The diversity of social behavior-inspired paradigms reflects the complexity and richness of the natural world. These paradigms, inspired by the collective behaviors of social animals or plants, offer unique perspectives and strategies for problem solving. However, as highlighted by the “no free lunch” (NFL) theorem, no single paradigm excels in all situations. We summarize the strengths and weaknesses of these paradigms in Table 1. Obviously, these methods inspired by different behaviors induce similar adaptive mechanisms to balance exploration and exploitation in optimization to improve algorithm performance. However, most paradigms still suffer from common shortcomings such as parameter sensitivity and limited theoretical explanation. This section aims to summarize the natural mechanisms and update rules of social behavior-based paradigms, thus providing a substantial contribution to the reduction of unreasonable “metaphors”.

**Table 1.** Strengths and weaknesses of mainly social behavior-inspired paradigms

Paradigm	Characteristics and strengths	Weakness
PSO [107]	1) Simple implement. 2) Few parameters. 3) Hybridization capability. 4) Fast convergence	1) Parameter sensitivity. 2) Population size impact. 3) Difficulty in dynamic environments. 4) Performance limits in very complex problems.
ACO [108]	1) Decentralized decision-making and dynamic adaptation. 2) Positive feedback mechanism. 3) Theoretical foundation in routing and scheduling.	1) Parameter sensitivity. 2) High computational demand for complex or large-scale problems. 3) Risk of suboptimal convergence.
ABC [109]	1) Resilience and diversity in search. 2) Good exploitation. 3) Dynamic adaptability.	1) Slower convergence in complex scenarios. 2) Exploration-exploitation challenge. 3) Parameter sensitivity
GWO [110]	1) Hierarchical decision-making. 2) Few parameters.	1) Slower in unimodal problems. 2) Suboptimal trapping in complex scenarios. 3) Struggles with dynamic environments.
MFO [114]	1) Strong convergence with spiral pattern. 2) Robust parameter changes due to the simple parameter and spiral movement.	1) Risk of stagnation. 2) Parameter sensitivity. 3) Ineffectiveness in discrete problems. 4) Difficulty with dynamic problems.
BA [115]	1) A good combination of major advantages features of PSO and harmony search. 2) High convergence rate in the early stages of the iterations. 3) Good exploitation ability	1) Risk of premature convergence the exploration and exploitation. 2) Parameter sensitivity. 3) Stagnation in the exploitation phase.
SOA [118]	1) Adaptive optimization behavior. 2) Diverse search mechanisms include spiraling flight for local search and straight flight for global search.	1) The exploration ability is relatively weak. 2) High computational cost. 3) Parameter sensitivity
MBO [119]	1) Wider variety maintained by dual population structures. 2) Robustness in multimodal problems.	1) Parameter sensitivity. 2) Convergence speed can be slower in simpler problems. 3) High computational cost.
PIO [121]	1) Fast convergence. 2) Effective in dynamic environments. 3) Simplicity in setup.	1) Risk of premature convergence. 2) Limited benchmarking data. 3) Scalability issues in high dimension
SBO [137]	1) Diversity maintenance. 2) Enhanced local search.	1) Limited testing and validation. 2) Complexity and slow convergence. 3) Parameter sensitivity.
MA [138]	1) Hybrid PSO-DE approach, leveraging the strengths of both. 2) Mating behavior encourages diversity. 3) Flexible adaptability.	1) Complexity. 2) Parameter sensitivity. 3) Computational overhead
BWO [139]	1) Diverse solutions. 2) Mitigating premature convergence. 3) Simplicity in implementation.	1) Newness and limited testing. 2) Critical tuning requirements. 3) Computationally intensive. 4) Scalability issues.
COA [140]	1) Diversity maintenance through territorial mimicry, social learning, and social hierarchy . 2) Collaborative search balance exploration and exploitation. 3) Good scalability.	1) Parameter sensitivity. 2) Complexity in design. 3) Computation overhead. 4) Limited empirical testing.
EHO [141]	1) Adaptability to dynamic environments. 2) Memory utilization. 3) Suitability for multi-modal problems	1) High computational complexity. 2) Parameter sensitivity. 3) Scalability challenges in complex scenarios.
CSO [142]	1) Dynamically switch between seeking and tracing modes. 2) Simplicity in setup. 3) Versatility in application.	1) Parameter dependency. 2) Premature convergence risk. 3) Limited recognition and support.
FA [143]	1) Automatic subgroup formation enhances robustness and efficiency. 2) Dynamically balance exploration and exploitation. 3) Robust to noise and user-friendly simplicity.	1) Risk of local minima traps. 2) Scalability concerns. 3) Parameter sensitivity.

#### Foraging behavior

Group foraging, also known as social foraging, is the process of finding, capturing, and feeding by a group of closely allied individuals, usually in the form of collective movements. The success of foraging relies on the individual itself and the group. In group foraging, members of groups such as flocks of birds, wolves, and elephants may benefit individual foragers by increasing foraging time, food contact rates, and capture efficiency and reducing the unit of time or energy expended of one individual [102,103].

The main factor that distinguishes the collective behavior of group foragers from that of individual foragers is group cohesiveness. Generally, in social foraging theory, the problem involves the fitness of an individual’s behavior, and the model is based on game theory and evolutionary stabilization strategies [104]. As noted by studying male rhesus macaques in [105], the marginal value theory (MVT) is the most appropriate for observing group foraging behavior. The MVT theory suggests that food resources in an environment are distributed in discrete patches, where the animal consumes food resources by feeding in patches and moving between patches. The animal is faced with diminishing returns as the food in a patch increases in time, so it has to choose the right time to leave to feed in the next patch. According to this theory, group foraging is a random rather than a deterministic process, and information is also “shared” between groups. In this process, individuals may be forced to search and forage, which is a dynamic exploitation of resources [106].

By studying the habits of a number of group foragers, and combining the group foraging characteristics described above, a number of swarm intelligence models based on group foraging behavior have been proposed, such as PSO [107], ant colony optimization (ACO) [108], artificial bee colony algorithm (ABC) [109], and grey wolf optimizer algorithm (GWO) [110]. These models typically cover the 3 phases of group foraging: locating and surrounding prey, hunting, and attacking, aiming at “information sharing” in a stochastic environment with dynamic “exploitation-exploration” to find the optimal solution [103].

Among these paradigms, the top 4 cited paradigms are PSO, ACO, ABC, and GWO. The relevant features are shown in Table 2. The parameter introductions related to Table 2 can be found in Tables 3 and 4.

**Table 2.** Classical paradigms based on foraging behavior

Name	Patterns	Main idea of update individuals	Typical application
Particle swarm optimization (PSO) [107]	Group foraging and information sharing	Velocity update: $v_{i,d}^{\left(t+1\right)}=w\cdot v_{i,d}^{\left(t\right)}+c_{1}\cdot rand_{1}\cdot \left(P_{\text{best},i}-x_{i,d}^{\left(t\right)}\right)+c_{2}\cdot rand_{2}\cdot \left(G_{\text{best}}-x_{i,d}^{\left(t\right)}\right)$ Position update: $x_{i,d}^{\left(t+1\right)}=x_{i,d}^{\left(t\right)}+v_{i,d}^{\left(t+1\right)}$	Influenza predicting [288] Parametric optimization [289] Scheduling problems [290] Energy system [291]
Ant colony optimization (ACO) [108]	Group foraging and trail pheromone	Edge selection: $P_{\mathrm{ab}}^{k}=\frac{{\left[\tau_{\mathrm{ab}}\right]}^{\alpha }\cdot {\left[\eta_{\mathrm{ab}}\right]}^{\beta }}{\sum_{c\in \text{allowe}d_{k}}‍{\left[\tau_{\mathrm{ac}}\right]}^{\alpha }\cdot {\left[\eta_{\mathrm{ac}}\right]}^{\beta }}$ Pheromone update: $\tau_{\mathrm{ab}}\leftarrow \left(1-\rho \right)\tau_{\mathrm{ab}}+\sum_{k}^{m}‍\Delta \tau_{\mathrm{ab}}^{k}$	Scheduling problems [292] Traffic congestion problems [293] Network routing [294]
Artificial bee colony algorithm (ABC) [109]	Group foraging waggle dance and social hierarchy	Employed bee phase: $x_{i,d}^{\left(t+1\right)}=x_{i,d}^{\left(t\right)}+\phi_{i,d}^{\left(t\right)}\cdot \left(x_{i,d}^{\left(t\right)}-x_{k,d}^{\left(t\right)}\right)$ Scout Bee Phase: $x_{i,d}^{\left(t+1\right)}=x_{d}^{\min}+\phi_{i,d}^{\left(t\right)}\cdot \left(x_{d}^{\max}-x_{d}^{\min}\right)$	Assignment problems [295] Image and video processing [296] Portfolio selection [297]
Grey wolf optimizer (GWO) [110]	Group foraging and social hierarchy	Encircling prey: $x_{i}^{\left(t+1\right)}={x_{p}}^{\left(t\right)}-A\cdot D$ $\left(D=\left|C\cdot {x_{p}}^{\left(t\right)}-x_{i}^{\left(t\right)}\right|\right)$ Hunting: $x_{i}^{\left(t+1\right)}=\frac{x_{\alpha }^{\left(t\right)}+x_{\beta }^{\left(t\right)}+x_{\delta }^{\left(t\right)}}{3}$	Feature selection [298] Power dispatch problems [299] Scheduling problems [300]

**Table 3.** Common parameter introduction in Tables 2, 5, 7, 9, and 12

Variable	Content
*x_i_*	Position of a agent *i*.
*d*	Dimension of a agent.
*t*	Number of iteration.
$x_{i,d}^{t}$	Position of agent *i* in dimension *d* at *t* iteration.
$v_{i,d}^{t}$	The velocity of agent *i* in dimension *d* at *t* iteration.
*c*	Cognitive and social acceleration coefficients.
*rand*	Random numbers are generated for each dimension and each agent at each iteration, typically from a uniform distribution in the range [0,1].
*e*	Base of the natural logarithm.
*l*	Random number in [−1,1].
*P*_*best*, *i*_	The personal best position of agent *i* has found so far.
*G_best_*	The global best position found by the entire swarm.
$x_{d}^{\max}$ and $x_{d}^{\min}$	Lower and upper bounds for the dimension.
*Lévy *	Lévy flight function.

**Table 4.** Parameter introduction corresponds to Table 2

Variable	Content
*w*	The inertia weight, which controls the influence of the previous velocity on the new velocity. It helps in balancing exploration and exploitation.
*τ_ab_*	Amount of pheromone on path (edge) (*a*, *b*).
*ρ*	Rate of pheromone evaporation (a constant between 0 and 1). This parameter controls the rate at which pheromone evaporates from the path. A higher value of *ρ* means faster evaporation.
*τ_ab_*	Heuristic information (e.g., inverse of the distance for TSP) for edge (*a*, *b*).
allowed*_k_*	The set of nodes that agent *k* can visit next.
*α*, *β*	Controlling parameters.
$\sum_{k}^{m}‍\Delta \tau_{\mathrm{ab}}^{k}$	The total pheromone deposited on the path between nodes *a* and *b* by all *m* agents.
$\Delta \tau_{\mathrm{ab}}^{k}$	The amount of pheromone deposited on the path by agent *k*. It is usually determined by the quality of the solution found by agent *k*.
Δ*τ_ab_* = *Q*/*L_k_*	Amount of pheromone deposited if agent *k* traveled edge (*a*, *b*), or 0 otherwise. *Q* is a constant; *L* is the length of the tour made by agent *k*.
$\phi_{i,d}^{\left(t\right)}$	A random number between [−1,1], controlling the perturbation and influencing the direction and magnitude of the change.
$x_{k,d}^{t}$	The current solution for a randomly chosen *k*th agent in the *d*th dimension at iteration *t*. The *k*th agent is selected randomly and must be different from the *i*th agent.
*x_p_*	The position of the swarm (or an estimated position based on the best solutions found so far) at iteration *t*.
*A*	A coefficient vector that influences the step size and direction of the wolves’ movement.
*D*	A coefficient vector that influences the oscillation behavior around the swarm.
*x_α_*, *x_β_*, *x_δ_*	Best 3 solutions obtained so far.

The PSO algorithm, the best-known biological-based paradigm, was originally proposed for modeling the social behavior of graceful but unpredictable movements of a flock of birds, where each agent serves as a collision-proof bird in flight. Essentially, simple social interactions are utilized to generate flock intelligence in the PSO algorithm [111]. According to Table 2 and Fig. 6A, the PSO update principle consists of 2 main components, velocity update and position update. The velocity of each particle $v_{i,d}^{\left(t\right)}$ is adjusted by considering its own best-known position (*P*_*best*, *i*_) and the best-known position among all particles in the group (*G_best_*). This update contains cognitive and social components, where cognitive refers to the particle’s own experience and social refers to learning from the group. After updating the velocity, the position of each particle $x_{i,d}^{t}$ is updated by adding its new velocity to its current position. This step moves the particle to a region of higher fitness in the search space. Overall, the PSO algorithm guides them toward optimal solutions based on their own experiences and the experiences of their neighbors in the swarm. The ACO algorithm is inspired by the foraging behavior of ants, which use pheromones to communicate and share information. Table 2 illustrates that the main function of ACO involves 2 main steps: (a) Edge selection: Each ant selects the next node to visit based on a probability that depends on the pheromone level and heuristic information. (b) Pheromone update: After all ants have completed their tours, pheromone levels on edges are updated to reflect the quality of the solutions found, with pheromone evaporation and deposition guiding future search efforts. The ABC algorithm models the nectar collection mechanism of bees by dividing the colony into employed bees, onlookers, and scouts following a social hierarchy. Table 2 shows that ABC uses employed and scout bee phases for updating solutions. Employed bees search for new solutions by exploring the neighborhood of their current positions. Scout bees introduce diversity by randomly generating new solutions when an employed bee’s solution cannot be improved further. This dual approach balances exploration and exploitation, effectively preventing stagnation.

Similar to the ABC, grey wolves also have a social hierarchy. According to Table 1, the update mechanism of GWO emulates grey wolves’ social hierarchy and hunting tactics. Wolves (agents) encircle prey (best solution) and update their positions accordingly. GWO categorizes wolves into alpha (*x_α_*, the best solution), beta (*x_β_*, the second-best), and delta (*x_δ_*, the third-best). The remaining wolves in the pack update their positions based on these 3 leaders.

#### Navigation behavior

Several species of animal in nature move in an orientated manner seeking food, avoiding predators, finding mates, homing, and navigating between areas essential to their survival. The role of navigation in animals is of great significance, as it involves the neural processing of sensory inputs, and the integration of different types of cues to orientate oneself and locate direction, aiming to guide toward their destination [112]. In 1873, Darwin [113] proposed that animals have the ability to navigate by dead reckoning, i.e., navigating by magnetic “compass” perception or by stars. From then on, researchers have investigated the mechanisms of how animals navigate based on senses. Existing relevant mechanisms and hypotheses have been proposed such as sun-based orientation, magnetism-based orientation, olfactory-based navigation, and cognitive map-based navigation. Generally, animals possess more than one orientation mechanism [112]. The researchers established prevalent optimization algorithms based on the navigation behavior, as shown in Table 5. The parameter introductions related to Table 5 can be found in Tables 3 and 6.

**Table 5.** Classical paradigms based on navigation and migration behaviors

Name	Patterns	Main idea of update individuals	Typical application
Moth flame optimization (MBO) [119]	Transverse orientation	Position update: $x_{i}^{\left(t+1\right)}=d_{\mathrm{ij}}\cdot e^{b\cdot l}\cdot \cos\left(2\pi l\right)+y_{j}$	Image recognition [301] Power dispatch [302] Engineering design [303]
Bat algorithm (BA) [115]	Echolocation and group foraging	Position update: $\left\{\begin{array}{cc} x_{i}^{\left(t+1\right)}=x_{i}^{\left(t\right)}+v_{i}^{\left(t+1\right)} & \\ v_{i}^{\left(t+1\right)}=v_{i}^{\left(t\right)}+\left(x_{i}^{\left(t\right)}-G_{\text{best}}\right)\cdot f_{i} & \end{array}\right.$ Frequency update: *f_i_* = *f_min_* + (*f_max_* − *f_min_*) · *rand* Local search: $x_{i}^{\left(t+1\right)}=G_{\text{best}}+l\cdot A^{t}$	Schedule problems [304] Tuning parameters [305] Feature selection [306] Multi-objective optimization [307]
Seagull optimization algorithm (SOA) [118]	Group migration and aggression	Migration: $x_{i}^{\left(t+1\right)}=\left|x_{i}^{\left(t\right)}+M_{i}^{t}\right|$ and $M_{i}^{t}=B\cdot \left(G_{\text{best}}-x_{i}^{\left(t\right)}\right)$ Attack: $x_{i}^{\left(t+1\right)}=x_{i}^{\left(t\right)}\cdot s_{x}\cdot s_{y}\cdot s_{z}+G_{\text{best}}$	Feature selection [308] Engineering design [309] Multi-objective optimization [310] Energy Modelling [311]
Monarch butterfly optimization (MFO) [119]	Migration	Migration operator: $x_{i,d}^{\left(t+1\right)}=\left\{\begin{array}{cc} x_{r1,d}^{\left(t\right)} & \;\text{if}\;\mathrm{rand}\cdot \text{peri}\leq p\\ x_{r2,d}^{\left(t\right)} & \;\text{else} \end{array}\right.$, Adjusting operator: $x_{i,d}^{\left(t+1\right)}=\left\{\begin{array}{cc} G_{\text{best}} & \;\text{if}\;\mathrm{rand}\leq p\\ x_{r3,d}^{\left(t\right)} & \;\text{if}\;\mathrm{rand}>p\leq \mathrm{BAR}\\ x_{i,d}^{\left(t\right)}+\alpha \cdot \left(\mathrm{Lévy}\left(x_{i}\right)-0.5\right)\left(x_{i,d}^{\left(t\right)}+1\right) & \;\text{if}\;\mathrm{rand}>p>\mathrm{BAR} \end{array}\right.$	Wireless sensor network optimization [312] Schedule problems [313] Energy management and conversion [314]
Pigeon-inspired optimization (PIO) [121]	Homing, map, and compass mechanism	Map and compass factor phase: $\left\{\begin{array}{ll} x_{i}^{\left(t+1\right)}=x_{i}^{\left(t\right)}+v_{i}^{\left(t+1\right)} & \\ v_{i}^{\left(t+1\right)}=e^{-R\cdot \left(t\right)}\cdot v_{i}^{\left(t\right)}+\mathrm{rand}\cdot \left(G_{\text{best}}-x_{i}^{\left(t\right)}\right) & \end{array}\right.$ Landmark factor phase: $\left\{\begin{array}{c} N=\left[N/2\right]\\ x_{\text{center}}^{t}=\frac{\sum_{i=1}^{N}‍x_{i}^{t}\cdot w\left(x_{i}^{t}\right)}{\sum_{i=1}^{N}‍w\left(x_{i}^{t}\right)},\\ x_{i}^{\left(t+1\right)}=x_{i}^{t}+\mathrm{rand}\cdot \left(x_{\text{center}}^{t}-x_{i}^{t}\right), \end{array}\right.$	Image process [315] Unmanned aerial vehicle (UAV) flocking control [316] Many-objective optimization problems [317] Control parameter design [318]

**Table 6.** Parameter introduction corresponds to Table 5

Variable	Content
*d_ij_*	Distance between *i*th moth and *j*th flame.
*b*	a constant for defining the shape of the logarithmic spiral.
*y_j_*	Position of *j*th flame.
*cos*(2*πl*)	A cosine function that introduces oscillation around the flame.
*A^t^*	Average loudness of all bats at iteration *t*.
*f_i_*	Frequency associated with the *i*th bat, influencing step size.
*f_min_* and *f_max_*	Minimum and maximum frequencies.
*B* = 2 · *A*^2^ · *rand*	Random number. It is responsible for balancing the exploration and production capacity of the algorithm.
$M_{i}^{t}$	The migration vector for the *i*th seagull at iteration *t*.
$\left\{\begin{array}{cc} s_{x}=s_{r}\times \cos\left(k\right) & \\ s_{y}=s_{r}\times \sin\left(k\right) & \\ s_{z}=s_{r}\times k & \\ s_{r}=u\times e^{\mathrm{kv}} & \end{array}\right.$	*s_r_* represents the radius of the spiral. *k* is a random number in [0, 2*π*]. *u* and *v* represent the constants of the helix shape.
*N*	Total number of a population.
peri	A period factor that determines the likelihood of migration.
$x_{r1,d}^{\left(t\right)}$ and $x_{r2,d}^{\left(t\right)}$	The position of 2 monarch butterflies randomly selected from subpopulation1 (*r1*) and subpopulation2 (*r2*) in the *d*th dimension at iteration *t*.
$x_{r3,d}^{\left(t\right)}$	The position of 2 monarch butterflies randomly selected from subpopulation2 (*r2*) in the *d*th dimension at iteration *t*.
BAR	A threshold value used in the adjusting operator to determine different adjustment behaviors.
*α*	A scaling factor used in the *Le* ′ *vy* flight term.
*p*	A probability threshold for determining migration or adjustment actions.
*R*	A constant that controls the rate of decay for the velocity.
$x_{\text{center}}^{t}$	The weighted average center position of all positions at iteration *t*.
$\begin{array}{l} w\left(x_{i}^{t}\right)=\left\{\begin{array}{ll} \frac{1}{f\left(x_{i}\left(t\right)\right)+\epsilon }, & \min\\ f\left(x_{i}\left(t\right)\right), & \max \end{array}\right. \end{array}$	*f* is the cost function value. *min* and *max* indicate minimization and maximization problems separately.
*ϵ*	An arbitrary nonzero constant.

**Fig. 7.**
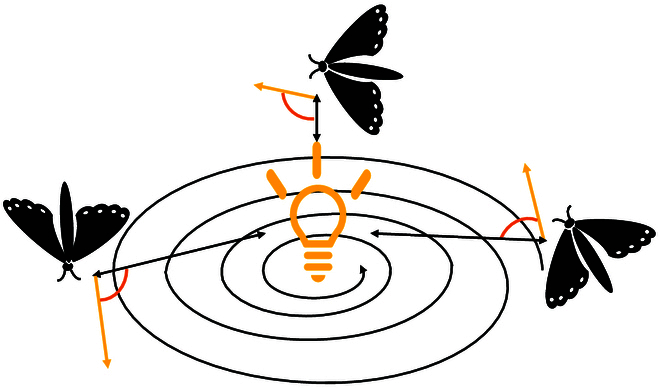
The moth moves in a spiral at the same angle as the light.

For both diurnal and nocturnal animals, celestial cues provide a wealth of information about orientation. A typical example is the moth. The moth has evolved to travel through night adopting a mechanism of transverse orientation to navigate. Since moonlight is at a great distance, this mechanism allows moths to fly in a spiral by maintaining a steady angle with respect to the moon. As shown in Fig. 7, in the case of artificial light, moths attempt to move in a spiral at the same angle as the light source. Inspired by the navigation mechanism of moths, Mirjalili [114] proposed the moth flame optimization (MFO) algorithm. According to Table 5, MFO first generates a random population of moths in the solution space and then calculates the fitness value of each moth to determine their positions. The best position found so far is marked by the flame. The algorithm relies on a spiral motion function to guide the moths toward the flame, updating their positions accordingly. This process is repeated iteratively, updating the positions of the moths and flames until the termination condition is satisfied, ensuring that the moths converge toward the optimal solution.

Bats are the only flying mammals. They make extensive use of echolocation. They emit loud pulses of sound and listen for echoes bouncing off their surroundings. Yang and He [115] presented a bat algorithm (BA) by modeling the dynamic behavior of bats with a type of ultrasound to detect prey and avoid obstacles. According to Table 5, bats in the algorithm have distinct positions, velocities, frequencies, loudness, and pulse rates. Initially, bats randomly navigate the search space, with frequency updates guiding velocity and movement per iteration, akin to echolocation. Frequency adjustments based on proximity to targets enable switching between exploration and exploitation. As bats near-optimal solutions, they reduce the loudness and increase pulse rates, similar to natural bat behavior when targeting prey. This behavior ensures that the bats focus on fine-tuning their positions as they approach the optimal solution.

Navigational behavior is the foundation of biological migratory activity. Studies have shown that seagulls establish an olfactory map with scent to navigate and migrate. Most of the seagulls migrate seasonally, heading in groups for rich food sources and habitats [116]. In addition, seagulls also attack migrating birds [117]. Dhiman and Kumar [118] proposed the seagull optimization (SOA) algorithm inspired by the migration behavior and attack behavior of seagulls. Migratory behavior affects global exploration ability, and aggressive behavior influences local exploitation ability. As shown in Table 5, the optimization steps of the SOA are primarily divided into 2 phases: migration and attack. During the migration phase, agents initially avoid collisions and then move. During the migration phase, agents initially avoid collisions and then move according to the global best position found so far. In the attack phase, seagulls exhibit spiral natural movements, thereby updating their positions.

Apart from seagulls, monarch butterflies are known for migrating on a large scale by using compass navigation. Wang et al*.* [119] established a monarch butterfly optimization (MBO) algorithm by modeling the migration behavior of monarch butterflies. In MBO, the position of each monarch butterfly represents a feasible solution. Each monarch butterfly will be distributed over 2 continents (“Land” and “Subland”) to search for the optimal solution through offspring migration and computing fitness. According to Table 5, the main update involves migration and adjusting the position of the butterflies. For the butterflies in the “Land”, the positions are updated using the migration operator. For the butterflies in the “Subland”, use Lévy flight behavior or other modes to update their location. The migration operator allows butterflies on the “Land” to update their positions based on a probabilistic mechanism, enhancing global exploration. The adjusting operator, incorporating Lévy flight behavior, enables butterflies on the “Subland” to adjust their positions, facilitating local exploitation and fine-tuning of solutions.

Homing refers to the ability of an animal to navigate back to its original location in an unfamiliar area, which is found in birds, salmon, burrows, and other animals. The pigeon is a well-known animal with homing behavior accomplished through navigation and is widely used in wartime communication, daily posts, etc. The pigeon forms a “navigation map” through mechanisms such as memorized visual landmarks, compass, and magnetic field [120]. During homing, the pigeon constantly evaluates its route to navigate in the correct direction through multiple mechanisms. Inspired by the homing of pigeons, Duan and Qiao [121] established a pigeon-inspired optimization (PIO) algorithm based on the map and compass mechanism of the pigeon homing behavior. PIO achieves the best position by constructing and updating the “map and compass operator” and “landmark operator” using the pigeons’ locations throughout the iterations. According to Table 5, when iteration $t\leq t_{\max}^{1}$, pigeons use the map and compass operator to update their positions, where $t_{\max}^{1}$ is a custom parameter. When iteration $t_{\max}^{1}<t\leq t_{\max}$, pigeons use the landmark operator to update their positions. This dual-phase update mechanism ensures that the pigeons effectively balance exploration and exploitation.

#### Parasitic behavior

Parasitism is the phenomenon when 2 organisms live together, one benefiting while the other suffers. The former is called the “parasite” and the latter the “host”, with the latter providing nutrients and shelter to the former. About 40% of known species are parasitic, and almost all animals are hosts to at least one parasite [122]. There are more than 400 types of parasites in humans [123]. The Red Queen hypothesis proposes that both hosts and parasites are constantly interacting, opposing, and fighting each other in a parasitic and antiparasitic struggle, with both co-evolving to produce diversity [124]. The way birds nest parasitically is a compelling example.

Bird brood parasite is a special breeding behavior whereby organisms lay their eggs in the nests of other birds, leaving the others to incubate and raise them. The most famous example of nest parasitism in birds is the cuckoo, which possesses a superb method in the art of deception. It removes one of the eggs laid by its host and then lays it itself. The main factor in the selection of phenotype for cuckoo parasitic eggs is the ability to successfully bypass host defenses, that is, careful matching by mimicking the pattern and color of the host egg, a skill that requires a high degree of accuracy to ensure success [125]. This process pays off after a while; the cuckoo’s eggs will hatch before the host’s, confusing the host into instinctively driving its own eggs out of the nest and thus increasing care for the cuckoo’s chicks. Cuckoo chicks are naturally cunning and will mimic the calls of their host chicks to gain more foraging opportunities. On the other hand, if a host recognizes a cuckoo’s eggs in its nest, either it throws them away or keeps its own eggs to build a new nest. Hence, the cuckoo must imitate the host’s eggs more accurately, while the host must improve its skills in identifying the parasitic eggs, which is called the struggle for survival [126].

Yang and Deb [127] developed a cuckoo search (CS) algorithm mimicking the egg-laying pattern of the cuckoo, incorporating the typical features of Lévy flight with a power-law pattern exhibited by the flight behavior of many animals and insects. In this algorithm, each cuckoo lays one egg (solution) at a time and dumps it in a random nest, and the best eggs will be taken to the next generation. The main idea of the update step in the algorithm is:

$$x_{i}^{\left(t+1\right)}=x_{i}^{\left(t\right)}+\alpha ⊕\mathrm{Lévy}\left(\lambda \right),$$ (1)

where for each cuckoo *i*, *x_i_* represents the nest position. *α* is the stepsize control quantity. Lévy(λ) is derived from Lévy flight function. Moreover, CS is widely applied in frequency control problems [128], traveling salesman problems [129], and energy power [130].

#### Competitive behavior

Darwin [38] emphasized the importance of competition as a universal principle of biology. Biological competition is a dynamic process, divided into intraspecific and interspecific competition. Intraspecific competition occurs when members of the same species compete for the same resource, and interspecific competition is probable to happen when individuals of 2 different species share a limited resource. Models such as the “competition Lotka-Volterra equation” mathematize the effects of dynamic interspecific competition on populations [131]. Generally speaking, competitive behavior among animals is often linked to aspects such as access to mates, food, territory, or valuable resources. Thus, animal competitive behavior is intricately woven into various aspects discussed in other parts of the “Biological-Based Paradigms” section.

Interestingly, competition is evident in plant species, particularly in weeds. Weeds are vigorous, aggressive growth habit plants that adapt rapidly to changes in their environment resulting in a threat to agriculture. The biodiversity of weeds provides plants with diverse characteristics to adapt and exploit opportunity spaces, and to adapt locally through natural selection. As population density increases, it becomes more difficult for species with lower fitness to survive.

Inspired by the competitive relationship between invasive weeds and the native plants, Mehrabian and Lucas [132] proposed an invasive weed optimization (IWO) algorithm to solve the solution for optimization through weed initialization, growth, reproduction, spatial distribution, and competition. The main idea of the update step in IWO is reproduction and spatial distribution. For each seed *i*, *S_i_* new seeds are generated, where *S_i_* is proportional to the seed’s fitness:

$$\begin{array}{c} S_{i}=\left\lfloor S_{max}-\left(\frac{S_{\text{max}}-S_{\text{min}}}{\text{max}\_gen}\right)\cdot gen\right\rfloor , \end{array}$$ (2)

where *S*_*max*_ and *S*_*min*_ are the maximum and minimum number of seeds, respectively, and *gen* is the current generation number. The seeds are dispersed using a normally distributed random number with a mean of zero and a standard deviation that decreases over generations:

$$\begin{array}{c} \begin{array}{c} \sigma_{gen}={\left(\frac{\text{max}\_\;gen-gen}{\text{max}\_\;gen}\right)}^{n}\cdot \left(\sigma_{\text{initial}}-\sigma_{\text{final}}\right)+\sigma_{\text{final}}, \end{array} \end{array}$$ (3)

where *σ*_*initial*_ and *σ*_*final*_ are the initial and final standard deviations, respectively, and *n* is a nonlinear modulation index. In summary, IWO is a powerful and flexible algorithm inspired by the colonizing behavior of weeds. IWO is typically employed in engineering design [133] and recommended system [134].

#### Reproductive behavior

Sexual selection is a mode of natural selection by which animals in nature often obtain the right to mate. Darwin [135] stated that sexual selection depends on the struggle between males for the right to approach females. There are 2 mechanisms of sexual selection: intra-sexual selection, which is a competition between members of the same sex (usually males) to obtain a mate, and inter-sexual selection, in which members of one sex (usually females) select members of the opposite sex [135]. For most species, mating takes place between 2 individuals of the opposite sex, and animal courtship behavior is also a manifestation of sexual selection. The presentation of animal courtship behaviors involves complex displays of dancing, vocalization, nesting, or fighting ability, designed to attract the attention of the opposite sex. Males in many species produce complex multimodal signals covering more than one sensory modality, such as a combination of tactile, visual, and auditory signals. Therefore, researchers have developed various algorithms inspired by reproductive behaviors, with some typical examples presented in Table 7. The parameter introductions related to Table 7 can be found in Tables 3 and 8.

**Table 7.** Classical paradigms based on reproductive behavior

Name	Patterns	Main idea of update individuals	Typical application
Satin bowerbird optimization (SBO) [137]	Reproductive and information sharing	Elitism: $x_{i,d}^{\left(t+1\right)}=x_{i,d}^{t}+\lambda_{d}\left(\left(\frac{x_{j,d}+x_{\text{elite},d}}{2}\right)-x_{i,d}^{t}\right)$ Mutation: $\left\{\begin{array}{c} \;x_{i,d}^{t+1}∼N\left(x_{i,d}^{t},\sigma^{2}\right)\\ N\left(x_{i,d}^{t},\sigma^{2}\right)=x_{i,d}^{t}+\left(\sigma \cdot \mathrm{rand}\right) \end{array}\right.$	Congestion management [319] Engineer design [320]
Mayfly algorithm (MA) [138]	Reproductive waggle dance	Nuptial dance phase: For male: $\begin{array}{c} \left\{\begin{array}{cc} v_{i,d}^{t+1}=v_{i,d}^{t}+c_{1}e^{-\beta r_{p}^{2}}\left(P_{\text{best},i}-x_{\text{male},i,d}^{t}\right)+c_{2}e^{-\beta r_{g}^{2}}\left(G_{\text{best}}-x_{\text{male},i,d}^{t}\right) & \\ x_{\text{male},i}^{t+1}=x_{\text{male},i}^{t}+v_{i}^{t+1} & \end{array}\right. \end{array}$ For female:$\begin{array}{c} \left\{\begin{array}{cc} v_{i,d}^{t+1}=\left\{\begin{array}{c} v_{i,d}^{t}+c_{2}e^{-\beta r_{\mathrm{mf}}^{2}}\left(x_{\text{male},i,d}^{t}-x_{\text{female},i,d}^{t}\right),\text{if }f\left(x_{\text{female},i,d}\right)>f\left(x_{\text{male},i,d}\right)\\ v_{i,d}^{t}+f_{l}\cdot r,\text{if }f\left(x_{\text{female},i,d}\right)\leq f\left(x_{\text{male},i,d}\right) \end{array}\right. & \\ x_{\text{female},i}^{t+1}=x_{\text{female},i}^{t}+v_{i}^{t+1} & \end{array}\right. \end{array}$ Mating:$\left\{\begin{array}{ll} x_{\text{offspring}1}=\lambda \cdot x_{\text{male}}+\left(1-\lambda \right)\cdot x_{\text{female}} & \\ x_{\text{offspring}2}=\lambda \cdot x_{\text{female}}+\left(1-\lambda \right)\cdot x_{\text{male}} & \end{array}\right.$	Training ANNs [321] Energy modeling [322]
Black widow optimization (BWO) [139]	Reproductive siblicide and pheromones	Mating (crossover):$\left\{\begin{array}{ll} x_{i}^{\left(t+1\right)}=\alpha \times P_{i}^{\left(t,f\right)}+\left(1-\alpha \right)\times P_{i}^{\left(t,m\right)} & \\ x_{i}^{\left(t+1\right)}=\alpha \times P_{i}^{\left(t,m\right)}+\left(1-\alpha \right)\times P_{i}^{\left(t,f\right)} & \end{array}\right.$	Image processing [323] Autonomous car-driver scheme [324]

**Table 8.** Parameter introduction corresponds to Table 7

Variable	Content
*λ_d_*	Determines the attraction power in the goal bower.
*x*_*j*, *d*_	The position of a selected bowerbird by the roulette wheel procedure in the *d*th dimension.
*x*_*elite*, *d*_	The position of the elite (best) bowerbird in the *d*th dimension.
$\sigma =R\cdot \left(x_{d}^{\max}-x_{d}^{\min}\right)$	The standard deviation controlling the spread of the mutation.
*α*, *β*	Fixed visibility coefficient.
*λ*	A random number or a crossover factor that controls the contribution of each parent to the offspring.
*r*	A random number following a specific distribution, which could be uniform or Gaussian.
*r_g_*	Cartesian distance between *x_i_* and *G_best_*.
*r_p_*	Cartesian distance between *x_i_* and *P_best_*.
*r_mf_*	Cartesian distance between male and female mayflies.
*f*(·)	Fitness function.
$x_{\text{male},i,d}^{t}$	Current position of the *i*th male mayfly in the *d*th dimension at iteration *t*.
$x_{\text{female},i,d}^{t}$	Current position of the *i*th female mayfly in the *d*th dimension at iteration *t*.
*xoffspring*	Position of the offspring.
*f_l_*	A scaling factor for the random component.
$P_{i}^{\left(t,m\right)}$	The position of the *i*th male parent at iteration *t*.
$P_{i}^{\left(t,f\right)}$	The position of the *i*th female parent at iteration *t*.

Male courtship behavior is common, for example, male satin blue gardeners construct gazebos to attract females. They compete by stealing other males’ ornaments and will destroy their gazebos [136]. Building on these observations, Moosavi and Bardsiri [137] proposed a satin bowerbird optimization (SBO) algorithm to model the courtship behavior of male satin blue garden birds. SBO uses the “gazebo” as a solution, which updates the gazebos with information sharing and destroys other gazebos for mutation. According to Table 7, SBO incorporates elitism and mutation to update solutions. Elitism preserves the best solutions akin to the most attractive bowers built by experienced male bowerbirds. Mutation mimics the unpredictable environmental factors affecting bower construction, ensuring a degree of robustness and adaptability in the algorithm by occasionally altering bower (solution) attributes.

Mayflies are another example, with most males gathering in swarms a few meters above the water to perform dances by moving in up-and-down patterns. Females fly into these swarms and mate with the aerial males. Mating may last only a few seconds, and when complete, the female drops her eggs to the surface. Inspired by this, Zervoudakis and Tsafarakis [138] proposed the mayfly algorithm (MA), where the position of each mayfly in the search space represents the potential solution, and the mayfly continuously adjusts its own position during flight to obtain the optimal solution. According to Table 7, the MA uses 2 key phases: nuptial dance (exploration) and mating (exploitation and crossover). The nuptial dance phase updates the position of the male mayflies and female mayflies with a different formula. In the mating phase, crosses between male and female mayflies produce new offspring (solution).

Female courtship behavior is less typical in nature, and the black widow is an example of this. Black widow spiders move in a linear and spiral pattern within a grid, marking certain points of the web with pheromones to attract males. They eat males in mating. Producing offspring also eat each other and may even eat their mothers. Inspired by the reproduction behavior of black widow, Hayyolalam and Kazem [139] developed the black widow optimization (BWO). Potential solutions are considered as black widow spiders, allowing healthy and strong individuals to survive. According to Table 7, BWO generally consists of 3 main processes: mating, cannibalism, and mutation. The mating process involves the crossover operation, cannibalism is related to the selection mechanism, and mutation introduces randomness and diversity.

#### Other behaviors

Beyond the social behaviors described above, there are still some popular paradigms that also model other characteristics and behaviors of species as shown in Table 9. The parameter introductions related to Table 9 can be found in Tables 3 and 10.

**Table 9.** Classical paradigms based on other biological behaviors

Name	Patterns	Main idea of update individuals	Typical application
Coyote optimization algorithm (COA) [140]	Social hierarchy and social condition	Social hierarchy and pack update: $x_{i}^{\left(t+1\right)}=x_{i}^{\left(t\right)}+c_{1}\cdot \left(x_{\text{alpha}}-x_{i}^{\left(t\right)}\right)+c_{2}\cdot \left(x_{p,\mathrm{avg}}-x_{i}^{\left(t\right)}\right)$	Energy modeling [325] Feature selection [326] Economic dispatch [327]
Elephant herding optimization (EHO) [141]	Tribal renewal and separation	Clan update: $x_{\mathrm{ci},k}^{\left(t+1\right)}=x_{\mathrm{ci},k}^{\left(t\right)}+\mathrm{rand}\cdot \left(x_{\mathrm{ci},\text{best}}-x_{\mathrm{ci},k}^{\left(t\right)}\right)$ Matriarch update: $x_{\mathrm{ci},\text{best},k}^{\left(t+1\right)}=x_{\text{center},\mathrm{ci}}^{\left(t\right)}\cdot \mathrm{rand},x_{\text{center},\mathrm{ci}}=\frac{1}{N_{\mathrm{ci}}}\times \sum_{k=1}^{N_{\mathrm{ci}}}‍x_{\mathrm{ci},k,d}$ Separating operator: $x_{\text{worst},\mathrm{ci}}^{\left(t+1\right)}=x_{d}^{\min}+\mathrm{rand}\cdot \left(x_{d}^{\max}-x_{d}^{\min}+1\right)$	Support vector regression [328] Energy-based localization [329] Distribution systems [330]
Cat swarm optimization (CSO) [142]	Search and stalking	Seeking mode: $x_{i}^{\left(t+1\right)}=x_{i}^{\left(t\right)}\cdot \left(1+\mathrm{rand}\cdot \mathrm{SRD}\right)$ Tracing mode: $\left\{\begin{array}{cc} v_{i}^{\left(t+1\right)}=v_{i}^{\left(t\right)}+c_{1}\cdot \mathrm{rand}\cdot \left(G_{\text{best}}-x_{i}^{\left(t\right)}\right) & \\ x_{i}^{\left(t+1\right)}=x_{i}^{\left(t\right)}+v_{i}^{t+1} & \end{array}\right.$	Clustering [331] Scheduling problem [332] Energy modeling [333]
Firefly algorithm (FA) [143]	Bioluminescence	Move toward more attractive fireflies: $x_{i}^{t+1}=x_{i}^{t}+\beta \left(r\right)\cdot \left(x_{j}^{t}-x_{i}^{t}\right)+\alpha \cdot \epsilon_{i}$	Scheduling problem [334] Noisy optimization [335] Industrial optimization [336]

**Table 10.** Parameter introduction corresponds to Table 9

Variable	Content
*x_alpha_*	Position of the alpha (the best solution found by the pack).
*x*_*p*, *avg*_	Average position of all coyotes in the pack.
*x*_*ci*, *k*_	Position of *k*th elephant in *ci*th clan.
*x*_*ci*, *best*_	Best elephant in the *ci*th clan.
*x*_*center*, *ci*_	Center position in the *ci*th clan.
*N_ci_*	Number of elephants in the *ci*th clan.
SRD	Seeking range of dimension, determines the local search range.
*β*(*r*) = *β*_0_*e*^−*γr*^2^^	Attractiveness function, which typically depends on the distance *r* between fireflies *i* and *j*. *β*_0_ is the initial attractiveness at *r* = 0 and *γ* is the light absorption coefficient.
*α*	Step size coefficient.
$x_{j}^{t}$	The position of the *j*th firefly at iteration *t*, which is more attractive (has better fitness) than the *i*th firefly.
*ϵ_i_*	A random vector drawn from a Gaussian distribution or uniform distribution.

The coyote optimization algorithm (COA) [140] models the social organization of North American coyotes and their living conditions. According to Table 9, the position of each coyote in the search space is updated based on its social condition and the social condition of other coyotes in its pack and neighborhood. The elephant herding algorithm (EHO) [141] mimics the herding behavior of elephants, specifically how they live in clans and use their wisdom to guide the herd. According to Table 9, each clan’s position (solutions) updates via the matriarch’s guidance (*x*_*ci*, *best*_). A portion of the least effective elephants become nomads, exploring solutions independently. The cat swarm optimization (CSO) algorithm [142] is based on the search and tracing behaviors of cats. CSO randomly classifies the cats into seeking and tracing modes according to a mixture ratio (MR). According to Table 9, in seeking mode, cats evaluate and randomly choose a new position within a defined seeking range. In tracing mode, akin to PSO, cats update their positions by tracking the velocity and location of the best solution. The firefly algorithm (FA) [143] models the behavior of fireflies that attract other individual fireflies by their luminous signals. According to Table 9, FA performs individual updates based on the attractiveness of brighter fireflies. Each firefly symbolizes a potential solution. Attractiveness correlates with the objective function value, guiding fireflies toward more promising solutions. Movement integrates a randomness factor, fostering exploration. Over time, fireflies gravitate toward the brightest (optimal) solution.

### Immune system

According to modern medicine, the immune function is a response to antigenic stimuli, which is expressed in the ability of the IS to identify itself and to eliminate non-self [144]. The IS serves as the defense system of organisms. Compositionally, the IS consists of immune organs, immune cells, and immune molecules. The immune organs such as the spleen and the thymus are responsible for the production of immune cells. Immune cells are cells involved in the immune response process, such as lymphocytes and phagocytes, while immune molecules are mostly substances secreted by immune cells, such as antibodies and complement [145,146].

The IS has a hierarchical defense mechanism, commonly divided into 3 layers: (a) physical, (b) innate immunity, and (c) adaptive immunity [145]. The first layer consists of physical and chemical barriers such as epidermal skin and mucous membranes. The second layer is the immune response generated by the innate IS, which allows a rapid response to a wide range of pathogens, such as phagocytosis and the complement system. Layers (a) and (b) are innate immune mechanisms present in all multicellular organisms and rely on germline-encoded receptors from the innate IS to recognize pathogens. Layer (c) employs variable antigen-specific receptors generated by gene fragment rearrangements. Adaptive immunity has been shown to be a major driver of selection for tumor suppressor gene inactivation [147]. Adaptive immunity occurs when the IS recognizes invading pathogens through a variety of responses. Following pathogen clearance, some of the immune cells may become memory cells and remain in the body for a long time.

**Fig. 8.**
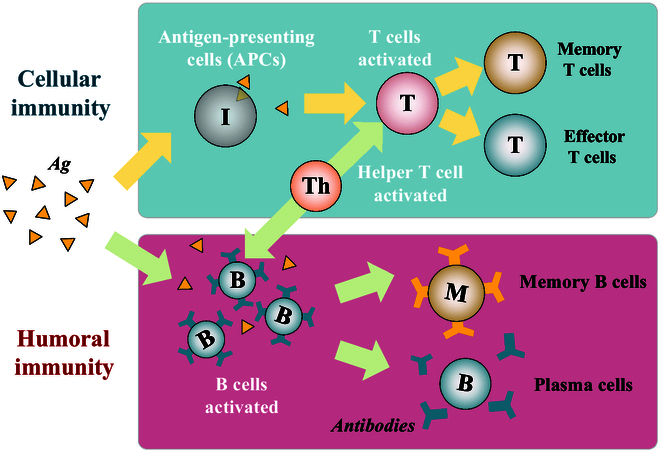
The adaptive immune response of the immune system.

The adaptive IS includes humoral and cellular immunity, with B lymphocytes primarily involved in the humoral immune process and T lymphocytes noted to be involved in the cellular immune process. As shown in Fig. 8, when being activated, some B lymphocytes and T lymphocytes become memory B cells and memory T cells, respectively. When the same pathogens reinvade, the adaptive IS rapidly produces a strong adaptive immune response [148]. Innate and adaptive immunity interact functionally in modern vertebrates. However, innate responses occur to the same extent regardless of the exposure to infectious agents encountered, whereas adaptive responses improve with repeated exposure to specific infections. Adaptive immunity can be acquired through natural infection or artificial vaccination. The genealogical relationship between memory and effector cells has profound implications for vaccine design and the development of effective T cell-based therapies.

### Artificial immune system

**Fig. 9.**
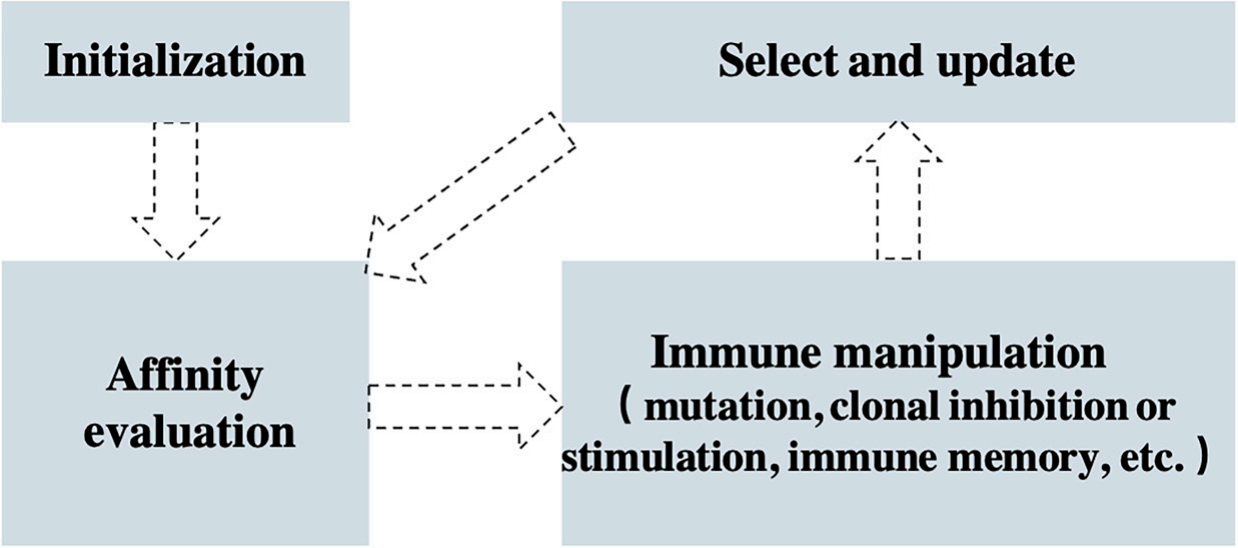
AIS usually considers the antigen as the problem to be solved and the antibodies as the potential solutions.

Biological ISs possess powerful information processing capabilities with feature extraction, learning memory, fault tolerance, and distributed properties [149,150]. Therefore, motivated by the biological IS, researchers have developed the AIS. The basic AIS models antigen recognition, memory, and self-regulation, which is akin to the biological IS. As shown in Fig. 9, AIS considers the antigen as the problem to be solved and the antibodies as the potential solutions.

Unlike fitness evaluation in EAs, AIS employs affinity as evaluation, including antibody–antigen affinity as well as antibody–antibody affinity. The affinity evaluation metric reflects the diversity of ISs. AIS is highly adaptable and robust. It is not sensitive to the setting of algorithm parameters or the quality of the initial solution. Moreover, centralized control is not required and can be processed in parallel, making AIS particularly suitable for multimodal optimization problems.

As shown in Table 11, AIS has been developed around 5 main theories of immunology: clonal selection theory, immune networks theory, negative selection theory, immune danger theory, and vaccination theory.

**Table 11.** Classical paradigms based on AIS

Biological mechanisms	Typical model	Typical application
Clonal selection theory	CLONALG [154]	Set covering problem [337], Scheduling problem [338]
Immune networks theory	Resource limited artificial immune system [156] AiNet model [157]	Classification problem [339], Disease detection [340]
Negative selection theory	Negative selection algorithm [159]	Intrusion detection systems [341], Fault detection system [342]
Immune danger theory	The dendritic cell algorithm [161]	Anomaly detection [343], Error detection [344]
Vaccination theory	Immune genetic algorithm [163]	Path planning [345], Anomaly detection [346]

**Fig. 10.**
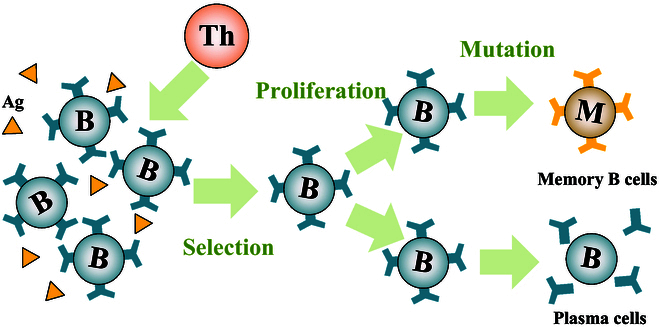
A simple diagram of clonal selection theory. It shows the cloning, proliferation, and mutation of B lymphocytes in response to antigen stimulation, and the generation of plasma cells as well as memory cells.

Clonal selection theory. Clonal selection theory explains the mechanism by which lymphocytes respond to specific antigens [151,152]. Figure 10 illustrates a simple diagram of clonal selection theory. Antibody-forming cell precursors, especially B cells, undergo T cell-dependent activation or T cell-independent activation and are clonally selected to produce antibodies. During this process, B cells undergo affinity maturation, a Darwinian evolution process characterized by B cell mutation and selection, which ensures that only those B cells producing high-affinity antibodies survive. These specialized cells then clone and differentiate into plasma cells and memory B cells. Plasma cells continuously secrete antibodies, making the IS more efficient at recognizing and clearing pathogens. Memory B cells have the unique ability to recognize previously encountered antigens, allowing for a more rapid and effective immune response upon reinfection [153].

The clonal selection algorithm is a class of algorithms obtained by mimicking the doctrine of clonal selection theory. A representative example is CLONALG [154]. CLONALG mimics the affinity maturation process in the immune response, which includes basic strategies such as selection, proliferation, and mutation.

Artificial immune network theory. Immune network theory attempts to explain how the adaptive IS regulates itself. The related work was mainly proposed by Niels Jerne in 1974. The immune network theory suggests that IS can maintain immune memory through a mutually reinforcing network of B cells. Additionally, antibodies, like other molecules, have antigenic epitopes that can be recognized by other antibodies, allowing for the distinction and regulation of different antibody types. The core idea of the theory holds that the elements of the IS (cells, antigens, antibodies, etc.) do not exist in isolation. They have a relationship of mutual stimulation, restraint, and recognition [150,155].

The artificial immune network algorithm is inspired by immune network theory. There are 2 main mainstream artificial immune network models, the resource-limited AIS (RLAIS) [156] and the AiNet [157]. RLAIS introduces the concept of an artificial recognition ball (ARB), which functions similarly to B cells. The AIS consists of a fixed number of ARBs that are stimulated by the primary stimulus *ps* of the antigen, the stimulus *nn* of the adjacent antibody, and the inhibitory *ns* of the adjacent antibody. The degree of antibody cloning is determined by the stimulus to the ARB. The AiNet models the immune network’s response to antigenic stimuli, incorporating processes such as antibody–antigen recognition, immune clone proliferation, affinity maturation, and network inhibition.

Negative selection theory. T cells recognize self and non-self antigens through T cell receptors (TCRs), which have a variety of structures resulting from gene rearrangements. Antigen-presenting cells (APCs) capture antigens and break them down into small peptides that interact with the TCRs. The affinity between antigens and TCRs, determined by their structures, controls T cell activation [158]. To prevent autoimmunity, T cells that recognize their own antigens engage in clonal deletion in the thymus [158]. Thus, T cells that recognize the “self” are eliminated, while those that do not recognize the “self” mature and are used to recognize the “non-self”.

Forrest et al*.* [159] developed a negative selection algorithm for anomaly detection based on positive and negative selection, which is similar to the process of “negative selection” that T cells undergo during maturation. In this algorithm, detectors are randomly generated, those detecting themselves are deleted, and those detecting non-self are retained for anomaly detection.

Immune danger theory. The core idea of the danger theory [160] is that the IS distinguishes between danger and safety by recognizing pathogens or signals from injured or stressed cells and tissues. Danger signals, which are crucial determinants of the immune response, activate APCs upon detecting damage. In contrast, healthy cells or cells undergoing normal physiological death do not emit danger signals. Any intracellular substance released from damaged or injured cells can act as a danger signal.

Dendritic cells, which are immune cells involved in antigen presentation, play a key role in this process. By integrating the immune danger theory into AIS, Aickelin and Greensmith [161] proposed a dendritic cell algorithm (DCA) based on the behavior of dendritic cells, which is widely used in intrusion detection problems. The algorithm generates a certain size population of dendritic cells and selects key attributes in the elements of the training set. These attributes are mapped to different types of signals, including security signals, danger signals, and pathogen-associated molecular pattern (PAMP) signals for solving intrusion detection problems.

Vaccination theory. Vaccines are biological therapies that provide adaptive immunity to specific infectious diseases. Modern vaccines usually contain components that resemble the disease-causing microorganism, such as weakened or killed microorganisms, their toxins, or a protein on their surface. The vaccine is administered to produce memory cells that generate antibodies corresponding to the pathogen. Additionally, T cells can destroy the virus’s ability to replicate by seeking out and destroying infected cells [162].

Inspired by this process, Jiao and Wang [163] proposed a new GA based on “immune vaccination”, namely, the immune genetic algorithm (IGA) that converges with probability 1. IGA constructs immunity operators through 2 main steps: a vaccination and an immune selection. The convergence speed of IGA is improved by introducing immunity operators to prevent the degradation of population diversity.

## Social-Cultural-Based Paradigms

Social-cultural-based paradigms are inspired by human social and cultural patterns of behavior. During the development of modern society, many behaviors containing human prior knowledge have been inherited. These prevalent social-cultural-inspired paradigms are detailed as shown in Table 12, including brain storm optimization (BSO), cultural algorithm (CA), imperialist competitive algorithm (ICA), and teaching-learning-based optimization (TLBO). The parameters related to Table 12 are shown in Tables 3 and 13.

**Table 12.** Classical paradigms based on social and cultural patterns of behavior

Name	Social and cultural patterns	Main idea of update individuals	Typical application
Brain storm optimization (BSO) [165]	Human interaction and cooperation	$\begin{array}{l} x_{i,d}^{\left(t+1\right)}=x_{\text{select},d}^{\left(t\right)}+\xi \cdot N\left(\mu ,\sigma \right)\\ \xi =\log\mathrm{sig}\left(\left(0.5\cdot T-t\right)/k\right)\cdot \mathrm{rand} \end{array}$	Power systems [347] Aerospace [348] Economics [166]
Cultural algorithm (CA) [168]	Evolution process of culture	$\begin{array}{l} x_{i}^{\left(t+1\right)}=x_{i}^{\left(t\right)}+\left|\left(x_{u}-x_{l}\right)\cdot \mathrm{rand}\right|,\text{if}\;x_{i}^{\left(t\right)}<x_{l}\\ x_{i}^{\left(t+1\right)}=x_{i}^{\left(t\right)}-\left|\left(x_{u}-x_{l}\right)\cdot \mathrm{rand}\right|,\text{if}\;x_{i}^{\left(t\right)}>x_{u}\\ x_{i}^{\left(t+1\right)}=x_{i}^{\left(t\right)}+\left(x_{u}-x_{l}\right)\cdot \mathrm{rand},\text{else} \end{array}$	Transportation engineering [349] Control [350] Feature selection [351] Scheduling problems [171] Design of skeletal structures [352]
Imperialist competitive algorithm (ICA) [169]	Phenomenon of imperialist competition	Assimilation process and revolution	Scheduling Problems [171] Design of skeletal structures [352]
Teaching-learning-based optimization (TLBO) [172]	Teaching process of a teacher to a learner	Teacher: $x_{i}^{\left(t+1\right)}=x_{i}^{\left(t\right)}+\mathrm{rand}\cdot \left(x_{\mathrm{tea}}^{\left(t\right)}-\text{round}\left(1+rand\right)\cdot \frac{1}{N}\cdot \sum_{i=1}^{N}‍x_{i}^{\left(t\right)}\right)$ Learning:$\begin{array}{c} \begin{array}{c} x_{i}^{\left(t+1\right)}=x_{i}^{\left(t\right)}+\mathrm{rand}\cdot \left(x_{i}^{\left(t\right)}-x_{\mathrm{rand}}^{\left(t\right)}\right),\text{if}\;f\left(x_{i}^{\left(t\right)}\right)<f\left(x_{rand}^{\left(t\right)}\right)\\ x_{i}^{\left(t+1\right)}=x_{i}^{\left(t\right)}+\mathrm{rand}\cdot \left(x_{\mathrm{rand}}^{\left(t\right)}-x_{i}^{\left(t\right)}\right),\text{else} \end{array} \end{array}$	Scheduling [353] Transformer fault judgment [174]

**Table 13.** Parameter introduction corresponds to Table 12

Variable	Content
*x*_*select*, *d*_	*d*th dimension of the individual selected to generate new individual.
*N*(*μ*, *σ*)	Gaussian random function with mean *μ* and variance *σ*.
*logsig()*	Logarithmic sigmoid transfer function.
*β*	Fixed visibility coefficient.
$\sigma =R\cdot \left(x_{d}^{\max}-x_{d}^{\min}\right)$	A variance.
*R*	A random number following a specific distribution, which could be uniform or Gaussian.
*T*	Maximum number of iterations.
*k*	For changing logsig() function’s slope.
*x_l_*, *x_u_*	Lower and upper bounds of the independent variable in excellent individuals, respectively.
*x_tea_*	Selected teacher individual.

### Brain storm optimization

Brain storm [164] occurs in human interaction and cooperation. When faced with a problem, a bunch of people with different knowledge backgrounds cooperate and communicate with each other. Then, the problem can be solved with high probability. Unexpected wisdom is born in this process. During the brainstorming process, there usually exists a facilitator, a brainstorming group, and several owners of problems that need to be solved. Facilitators force the brainstorming group to generate ideas based on certain principles. The problem owner selects better (and noteworthy) ideas from the set of ideas generated. Inspired by the brainstorming process, BSO [165] mainly consists of clustering and mutation. Through the convergence and divergence operations, the individuals in the population are grouped and diverged in search space. The optimal solution is searched during the aggregation and dispersion process. Because brainstorming organically combines swarm intelligence and data mining, it has been widely used in power systems, aerospace, economics [166], and other fields.

### Cultural algorithm

Culture [167] is a system of conceptual phenomena socially and historically encoded within and between groups of symbols. In recent years, some works have modeled the process of cultural evolution from both the perspectives of micro-evolution (the transmission of behavior or traits among individuals in a group) and macro-evolution (the formation of generalized beliefs based on personal experience). These generalized beliefs are used to constrain the behavior of individuals in related groups. The cultural system of dual inheritance described above supports the transmission of information at the individual and group levels. Inspired by the evolution process of culture, a 2-layer evolutionary mechanism consisting of population space and belief space is designed in the CA [168]. Population space models the evolution process of biological individuals according to certain behavioral rules from a microscopic perspective, and belief space models the evolutionary process of cultural formation, transmission, and comparison from a macroscopic perspective. The 2 spaces relate to each other based on the communication protocol, which effectively extracts and manages the evolutionary information.

### Imperialist competitive algorithm

**Fig. 11.**
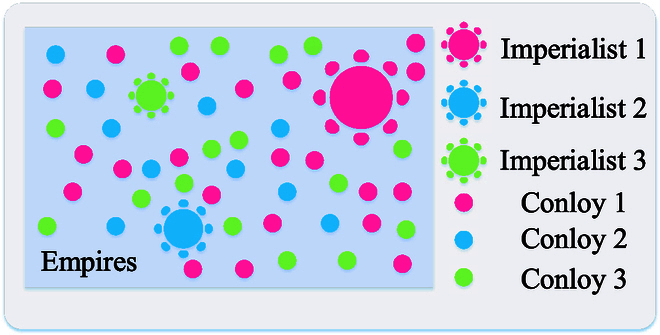
In the ICA, the best countries (with lower costs) were picked as imperialist countries, while the rest as colonies.

Beginning in 1970, the developed countries tried to dominate the less developed countries politically and militarily to expand their power and plunder their resources, which is called modern colonialism in history. The competition between imperialists entails the prosperity and advancement of the dominant national economy. To facilitate the dissemination of their values, the infrastructure of the colony was further built [169]. From an optimization perspective, the phenomenon of imperialist competition can be explained as follows: Colonies are lifted out of the valley (current position) and pushed to the peak of imperialism (new minima). The new status of the colonies could be better than imperialism at any time. The movement of the economic axis meant that the colonies improved their economic conditions by being influenced by the imperialist economy [170]. In ICA, those of the best countries (lower cost) are chosen as imperialist countries and the rest are treated as colonies, as shown in Fig. 11. After colonies were carved up by the imperialist states, they moved toward their associated imperialism in the space of the cultural state. That is to say, whether an empire survives or not hinges on its ability to undertake colonies from other competitors. Great empires grew in strength, while weaker empires crumbled. ICA has been broadly used in scheduling problems [171].

### Teaching-learning-based optimization

The TLBO [172] is inspired by the wisdom of the classroom teaching process, which mimics the teaching process of a teacher to a learner in a class. The improvement of the level of the students requires the teacher to “teach”. Students need to “learn” from each other to promote the absorption of knowledge at the same time. During the teaching process [173], learners are viewed as points distributed in the decision space, corresponding to a population of evolution-inspired paradigms. The best-performing students are defined as teachers of the class. A teaching phase and a learning phase are designed in the TLBO. The learner not only improves his own knowledge level by learning from the teacher in the teaching stage to improve the average knowledge level of the class but also randomly learns from other learners in the learning stage to broaden his knowledge level. The TLBO has been applied to many industrial fields such as scheduling and transformer fault judgment [174].

### Others

In addition to the above approaches, there is a range of paradigms inspired by culture and society. An important point of Confucius is that moderation is the best rule. Criss-crossing [175] is a new search paradigm inspired by the Confucian doctrine of the mean and the crossover operation in GAs. Many aspects of the universe are governed by duality, which refers to 2 opposing forces or conflicting states of nature at work. In Chinese philosophy, this idea is described as “yin” and “yang”, 2 complementary and interdependent extremes. One aspect gradually changes the other. This process is repeated until the balance of these 2 aspects produces harmony. Yin-yang pair optimization [176] is inspired by this idea to expect to balance the relationship between exploration and exploitation in optimization. The ultimate goal of a business hierarchy is to accomplish business-related tasks in the best possible way. Heap optimization [177] employs the heap structure to model the hierarchical structure of the company. The concept of the heap is adopted to form interactions between individuals. Three mathematical models are constructed for new individuals. Humans are great imitators or followers when solving any task. Group-solving skills are more effective than individual-solving skills when developing and exploring given problems. Social group optimization [178] is inspired by this idea so that each person enhances their knowledge by communicating with others in the group and learning from the best in the group. The above methods all hope to provide more advanced tools to solve practical problems by leveraging the phenomena in society and culture.

## Science-Based Paradigms

**Fig. 12.**
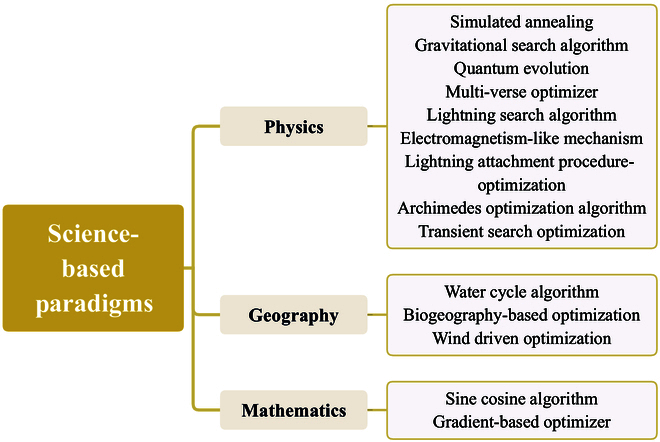
Science-inspired paradigms are divided into 3 areas: physics, geography, and mathematics.

Science-based paradigms are inspired by proven scientific theorems. This formulated knowledge comes from a variety of disciplines including natural, social, and formal sciences. These science-inspired paradigms are described in detail in this section, mainly in 3 areas: physics, geography, and mathematics as shown in Table 14 and Fig. 12.

**Table 14.** Classical paradigms based on science

Name	Mechanisms	Typical application
Simulated annealing (SA) [179]	Solid annealing	Image processing [180], Production scheduling [181], Machine learning [182,287]
Gravitational search algorithm (GSA) [184]	Newton’s law of universal gravitation	Power engineering [185], Control systems [183]
Quantum evolution (QE) [186]	Quantum computing [159]	Combinatorial optimization [354], Music composition [355], Cloud computing [356]
Multi-verse optimizer (MVO) [193]	Big Bang theory	Cloud computing [183,185], Scheduling [357], Pattern recognition [194]
Lightning search algorithm (LSA) [195]	Lightning discharges	Control systems [358,359], Wind farm layout [360], Image segmentation [196]
Water cycle algorithm (WCA) [206]	Hydrological cycle	Combinatorial optimization [207], Landslide prediction [208]
Biogeography-based optimization (BBO) [209]	Biogeography	Feature selection [210], Cloud computing [211], Shop floor scheduling [212]
Wind Driven Optimization (WDO) [213]	Flow of the Atmosphere	Electromagnetics [214], Image segmentation [215]
Sine Cosine Algorithm (SCA) [216]	Sine and Cosine	Feature selection [217], Parameter evaluation [218], Energy scheduling [219], Image processing [220]
Gradient-based Optimization (GBO) [221]	Gradient-based Newton’s Method	Parameter identification [222], Model design [223], Human activity recognition [224]

### Physics

**Fig. 13.**
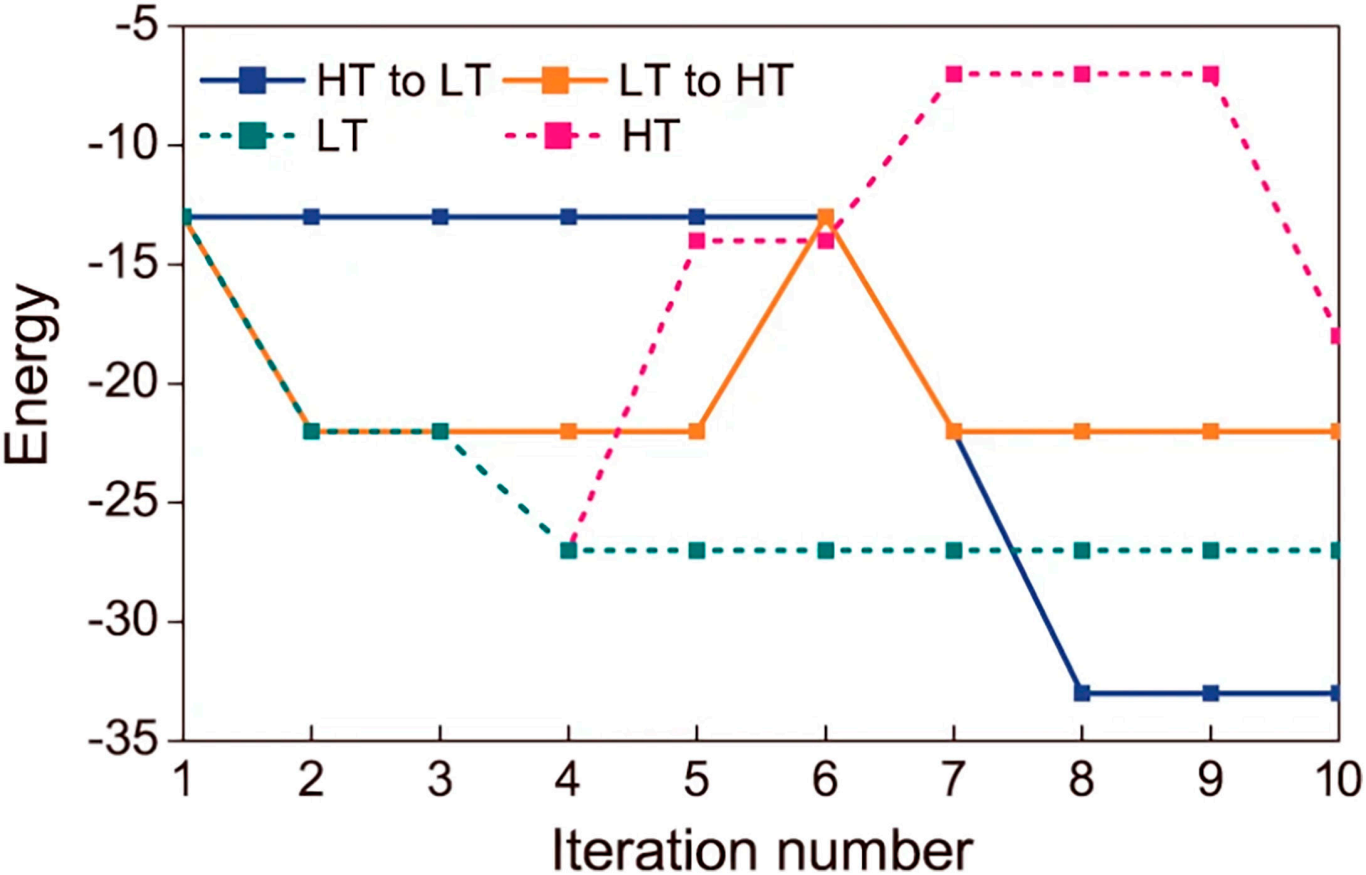
The performance of SA with 4 different strategies for optimizing the Boltzmann machine, including high *T_eff_* to low , low *T_eff_* to high *T_eff_*, low *T_eff_*, and high *T_eff_* (image from [287]).

Simulated annealing (SA) [179] is derived from the principle of solid annealing. The solid is heated to a sufficient height and then slowly cooled. During heating, the internal particles of the solid become disordered as the temperature rises. The internal energy increases. Then, particles tend to be ordered gradually, reaching an equilibrium state at each temperature. Finally, the particles achieve their ground state at room temperature. Moreover, their internal energy is minimized. The slow cooling implemented in the SA algorithm is explained as that the probability of accepting a worse solution slowly decreases as the solution space continues to be explored. SA algorithm is a general optimization algorithm. In theory, the SA algorithm can find the global optimal solution by probability. It has been widely employed in engineering, such as image processing [180], production scheduling [181], and machine learning [182]. Recently, SA has been used to optimize the tin oxide/MoS_2_-based Boltzmann machine [183] as shown in Fig. 13. By adjusting the value of *T_eff_*, different “cooling” strategies can be obtained. The figure shows the performance of 4 different strategies for optimizing the Boltzmann machine, including high *T_eff_* to low *T_eff_*, low *T_eff_* to high *T_eff_*, low *T_eff_*, and high *T_eff_*.

Gravity refers to the tendency of objects to accelerate each other. Each particle of the universe gravitates to others. Since gravity is everywhere, it makes it unique from other natural forces. Newton’s law of universal gravitation implies that gravity works among the separated particles with no intermediary or delay. Every particle attracts other particles with gravity. Inspired by this, the search agent for the gravitational search algorithm (GSA) [184] is a set of masses based on the interaction of Newton’s laws of gravity and motion, which mainly guides the motion of each particle according to Newton’s law of universal gravitation between 2 objects. The gravitational force is proportional to the mass of 2 particles and inversely proportional to the distance between them. GSAs have been widely used in power engineering [185] and control systems [183].

Inspired by quantum computing, quantum evolution (QE) [186] employs qubits to encode chromosomes, which mainly includes quantum chromosome observations and quantum gate updates. Quantum revolving gates, which play a critical role in QE, are the most commonly used quantum gates in this process. Because of the significance of revolving gates, they have received extensive attention in QE [187]. The simplicity and scalability of the QE structure have made it popular to integrate with various other heuristics, including PSO [188], immune cloning algorithms (ICA) [189], SA [190], and so on. Recently, Chai et al. [191] proposed the shortcuts to quantum approximate optimization algorithm (S-QAOA), which is an ideal choice for solving combinatorial optimization problems using current noisy quantum computers.

The Big Bang theory states that the universe began with a massive expansion known as the big bang [192]. Related to this hypothesis is that the big bang is the foundation of everything in nature. The multi-verse optimizer (MVO) [193] is modeled based on the principle that matter in the universe is transferred from white holes to black holes through wormholes. In the random creation process of the universe, objects with high expansion rates always tend to objects with low expansion rates due to gravitational effects. This gravitational effect can make objects transfer. According to the relevant cosmological rules, objects can gradually tend to the optimal position in the search space. MVO has been successfully implemented in several areas, such as cloud computing, scheduling, and pattern recognition [194].

The probabilistic and tortuous nature of lightning discharges originates from thunderstorms. Cloud-to-ground flashes are the most investigated occurrence in lightning research. During thunderstorms, a strong electric field is generated, which triggers an electron avalanche. In this case, it causes the negative coronal streamers and produces a current wave. As the current wave reaches the tip of the new leader, the coronal belt erupts and propagates outward, leading to the formation of a new space leader. This random growth process repeats continuously. Inspired by the phenomenon of lightning, the lightning search algorithm (LSA) [195] proposes 3 types of projectiles: transition projectiles for generating the initial leader population, space projectiles that attempt to become leaders, and lead projectiles representing the best solutions. A random distribution function based on the discharge probability characteristics and tortuosity characteristics of 3 projectile types is designed in LSA. LSA has been successfully applied to control systems, wind farm layout, and image segmentation [196].

In addition to the above methods, a series of paradigms based on physical principles have been proposed. Inspired by the attraction–repulsion mechanism between charged particles in an electromagnetic field, Birbil et al. proposed an electromagnetism-like mechanism algorithm [197], which has been applied to problems such as feature selection and flow shop scheduling [198]. The lightning attachment procedure consists of 4 important stages: air breakdown on the cloud surface, downward movement of the lightning channel, upward leading propagation from the ground, and final jump. Inspired by this process, lightning attachment procedure optimization [199] has been successfully used to solve problems such as power generation scheduling [200] and intrusion detection [201]. Inspired by Archimedes’ principle, Hashim et al*.* [202] presented the Archimedes optimization. It mimics the principle of buoyancy exerted on an object that is partially or fully immersed in a fluid. The buoyancy is proportional to the weight of the displaced fluid. It has been used in industrial design [203]. Transient search optimization [204], inspired by the transient behavior of switching circuits containing storage elements such as inductors and capacitors, has been applied in parameter estimation [205].

### Geography

Rivers or streams are formed when water flows from one place to another, typically moving downhill until they reach the sea. Water evaporates from rivers, lakes, and plant leaves (through transpiration), forming clouds in the atmosphere. These clouds condense and release water back to Earth as rain or precipitation, completing the hydrological cycle (water cycle). Based on observations of the water cycle process, Eskandar et al*.* [206] proposed the water cycle algorithm (WCA), which has been widely used in combinatorial optimization [207] and landslide prediction [208].

Biogeography-based optimization (BBO) [209] is inspired by the principles of biogeography. Through the constant migration and drift of species between regions, nature finally reaches a state of equilibrium. The update mechanism for BBO relies mainly on 2 operations: migration and mutation. Migration is modeled as statistical models such as linear, cosine, quadratic, and exponential. These models describe the process of “the more the number of organisms in a place, the lower the in-migration rate and the higher the out-migration rate”. The mutation process in BBO is analogous to that in GAs. BBO has been applied to problems such as feature selection [210], cloud computing [211], and shop floor scheduling [212].

In the atmosphere, winds flow to balance the air pressure. Specifically, it blows from a high-pressure region to a low-pressure region at a rate commensurate with the pressure gradient. When the air is in equilibrium and horizontal motion is stronger than vertical motion, the wind can be regarded as horizontal motion. The wind driven optimization (WDO) [213] is inspired by the flow of the atmosphere, where the movement of the wind can automatically compensate for the imbalance in atmospheric pressure. According to Newton’s second law, the motion law of a very small air unit is described. The final flow position of the air unit is used as a candidate individual to complete the modeling and solution of the problem. The velocities and positions of wind-controlled air masses in the WDO are renewed based on the physical equations governing atmospheric motion. WDO has been widely used in electromagnetics [214] and image segmentation [215].

### Mathematics

The optimization process of a sine cosine algorithm (SCA) [216] is divided into 2 stages. In the exploration phase, the algorithm identifies a feasible region in the search space by combining random solutions from the set of candidate solutions. In the exploitation phase, these random solutions are gradually refined. The rate of change in the exploitation phase is lower than that in the exploration phase. In SCA, multiple initial random candidate solutions are generated, which fluctuate outward or in the direction of the optimal solution based on the mathematical model of sine and cosine. This approach enables the algorithm to explore different regions in the search space effectively. SCA has been successfully used in feature selection [217], parameter evaluation [218], energy scheduling [219], and image processing [220].

Gradient-based optimization (GBO) [221] is inspired by gradient-based methods, particularly Newton’s method. The method mainly uses 2 kinds of operators: gradient search rule (GSR) and local escaping operator (LEO). A set of vectors is employed to explore the search space, with the GSR utilizing a gradient-based approach to accelerate convergence and achieve better positions within the search space. The LEO helps the algorithm escape local optima, enhancing its overall effectiveness.. Due to its simple structure, GBO has been widely used in various engineering optimization problems, such as parameter identification [222], model design [223], and human activity recognition [224]

## Challenges and Potential Future Research Directions

**Fig. 14.**
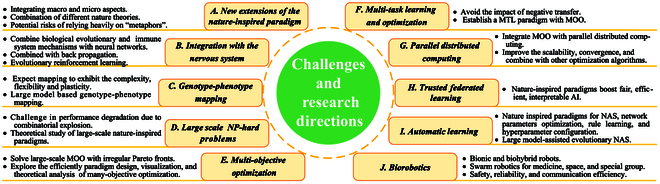
Challenges of the nature-inspired intelligent computing paradigm and potential future research directions.

Although the nature-inspired intelligent computing paradigm originated from diverse theories, it has developed its own unique trends over the decades. Nature-inspired intelligent computing paradigm presents innate advantages in solving real-world problems due to their parallelism, ease of expansion, and nonlinearity. The development of nature-inspired intelligent computing paradigms has moved forward in many research directions and differs from initial theories [13]. By reviewing classical nature-inspired intelligent computing paradigms, we summarize the top 10 potential future research directions and challenges, as shown in Fig. 14.

### New extensions of the nature-inspired intelligent computing paradigm

Most of the existing nature-inspired intelligent computing paradigms primarily focus only on the evolutionary and phenological behaviors of organisms, often neglecting the growth and developmental processes of organisms, and fail to bridge the link between the macro and the micro. For example, while clustered regularly interspaced short palindromic repeats (CRISPR) gene-driven evolutionary dynamics are noted in [225], similar models are largely absent in current evolutionary computations.

Combining different nature theories has become a popular topic. For example, through the integration of Lamarckianism and Darwinism [226], Lieberman et al*.* [227] combined evolutionary dynamics with graph representation. In addition, new rational mechanisms are constantly being explored and discovered to provide a source of new paradigms. Among group behaviors, Harpaz et al*.* [228] explain schooling behaviors in zebrafish, which converts visual input from neighbors into motor decisions. As scientific research progresses, more “empirical phenomena” will be explained, pointing toward new extensions of nature-inspired intelligent computing paradigms with theoretical implications.

However, it is crucial to recognize the potential risks of over-reliance on “metaphors”. “Metaphors” refers to paradigms in which there are unreasonable connections between natural mechanisms, mathematical models, and nature-inspired intelligent computing paradigms. Such weak or misleading connections can lead to the unnecessary reinvention of mechanisms and impede our understanding of these paradigms [30–33]. Therefore, it is more important to clearly delineate the relationship between natural mechanisms and paradigms to substantially reduce the reliance on “metaphors”.

### Integration with the nervous system

Although different in function, the nervous and ISs work in specific ways to coordinate and regulate the function of the whole organism, working together to maintain the homeostasis of the internal environment. The variety of these systems does not operate independently of each other. For example, intestinal immune cells allow movement to reduce inflammation in the central nervous system [229], and the neuronal signals control the innate IS [230]. Recently, Koren et al*.* [231] indicated that the brain stores and retrieves specific immune responses, extending the classical concept of immune memory to neuronal representations of inflammatory information. This finding underscores the inseparable connection between the brain and the IS, providing a stronger theoretical basis for immune computation.

Researchers have abstracted the neuronal structure of the nervous system into deep neural networks and built biological neural networks [232–234]. A typical example is solving the temporal credit-assignment problem (TCA) by exploring the characteristics of spiking neurons. Gütig [232] developed an aggregate-label learning (AL) rule based on the responses of spiking neurons to the TCA problem. The derivatives of these responses indicate the most rapid changes in neuronal activity, enabling spiking neurons to match their output spikes with the number of clues or features present in the input data. Building on this, Qin et al*.* [233] introduced an innovative attention-based loss function to solve the TCA problem. This function effectively integrates global temporal dynamics with intricate spike cluster data, thereby significantly enhancing the TCA capabilities of spiking neural networks (SNNs). In addition, several works combine evolutionary mechanisms with neural networks, such as the framework for evolutionary artificial general intelligence (FEAGI) [235], evolving probabilistic SNN (epSNN) [236], and EevoSpike NeuCube architecture [237].

In deep learning, backpropagation is commonly used to approximate. However, it is unlikely that the brain uses only backpropagation. Therefore, it is essential to “go back” to the biological sciences to uncover more meaningful evolutionary learning mechanisms to complement backpropagation mechanisms. Evolutionary RL is also a promising direction for developing deep neural networks that adapt to the complex environment [2,238,239].

### Genotype–phenotype mapping

The genotype–phenotype mapping plays a crucial role in the design of EAs. This mapping is the process of mapping genes toward their biology functions [13]. In algorithms, genotype–phenotype mapping refers to the process of identifying the relationship between a system’s hidden variable (genotype) and a measured observable quantity (phenotype) [240]. Research [241] noted that the development of animal appearance phenotypes is controlled by large gene regulatory networks. Existing evolutionary-inspired paradigms lack expression of the complexity, flexibility, and plasticity of biological organs and ecosystems [13].

Traditional evolutionary paradigms typically employ a simple, direct genotype-to-phenotype mapping. However, the emergence of indirect coding leads evolutionary-inspired paradigms to deal with more complex systemic problems. As a result, how to better optimize the “genotype-expression” representation is also a topical and challenging issue for future evolutionary-inspired paradigms [242].

In recent years, large models have shown the potential to achieve general AI [243]. These models’ powerful representation capabilities present new opportunities for genotype–phenotype mapping. Traditional genotypes, often carefully designed based on expert knowledge, have limited generalization performance [13]. Multimodal information such as natural language and vision can be directly used to describe problem characteristics. Directly searching for this information by vision and language models may provide a feasible way to achieve general optimization [84,244]. Therefore, developing novel nature-inspired intelligent computing paradigms based on language and visual representations is a promising direction.

### Large-scale problems

In reality, many physical world problems can be formulated as nondeterministic polynomial problems (NP-hard problems), such as transportation, industrial design, and data mining. Existing approaches to NP-hard problems are roughly categorized into 2 broad categories: exact algorithms and approximate algorithms. Exact algorithms, attempting to find the global optimal solution, consist of methods such as the branch-and-bound and the branch-and-cut. The nature-inspired intelligent computing paradigm provides a way to approximate the solution of NP-hard problems [245]. However, as the problem dimensionality increases, the problem complexity usually increases with the problem size, as well as the solution space of the problem grows exponentially with it. This results in a combinatorial explosion, and the performance of most available optimization algorithms degrades rapidly.

**Fig. 15.**
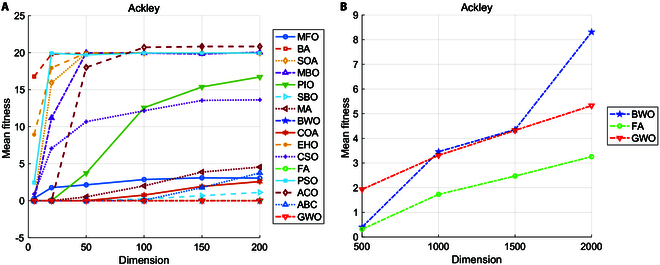
Trend of the average fitness value (smaller is better) with problem scale.

To further illustrate that large-scale problems are an important future research direction, we test the performance of popular biological-based paradigms in Table 1 on the benchmark problem Ackley at different problem scales. Figure 15A shows the average fitness value as a function of problem size. The *x* axis refers to the dimension of the decision variable, which varies from 5 to 200. It is obvious that the performance of all algorithms decreases as the problem size increases. To further reflect the performance changes of BWO, FA, and GWO, Fig. 15B shows the performance change curve at a larger problem scale (500 to 20,000). Experimental results still illustrate that these algorithms suffer performance collapse when dealing with large-scale problems. In the future, the applicability of the nature-inspired intelligent computing paradigm to large-scale problems should be further theoretically investigated.

Although nature-inspired intelligent computing paradigms are relatively efficient in obtaining the “best” solution through a search strategy rather than the exact approaches, they are still computationally expensive compared to traditional optimization methods such as Newton’s method and hill climbing. Therefore, how to reduce costs through surrogate models, cooperative coevolution algorithms (CCPSO [246], MLCC [247]), and other theoretically guaranteed methods are also promising research topics for future research [19].

### Multi-objective optimization

Multi-objective optimization (MOO) problems are broadly found in real-life applications such as recommendation systems, schedule cost problems, and scheduling decisions [248]. In MOO, decision-makers need to optimize multiple tasks simultaneously. The trade-offs between different objectives result in a set of incomparable Pareto-optimal solutions that do not dominate each other. When confronted with MOO problems with more than 4 objectives, the majority of methods suffer from severe performance degradation. This is due to the increased computational cost of evaluating the objective function, as well as the rapidly increasing number of nondominated solutions breaking the Pareto selection pressure [249]. Therefore, it is important to further explore the algorithm design [250], visualization [251], and theoretical analysis [252] of many-objective optimization problems. In addition, it is also challenging to solve large-scale MOO problems with irregular Pareto fronts [248].

### Multi-task learning and optimization

Multi-task optimization (MTO) aims to solve multiple optimization tasks simultaneously by exploiting the commonalities and differences between tasks [253]. In practical applications, related optimization tasks are ubiquitous. The population-based paradigm has natural implicit parallelism, which is highly compatible with MTO. So MTO has gradually flourished in the field of evolutionary computing in recent years [254]. However, with the increase in the number of optimization tasks, avoiding the impact of negative transfer remains an open challenge [255,256].

Multi-task learning (MTL) is considered to reflect the human learning process more accurately than single-task learning [257]. MTL aims to solve multiple learning tasks simultaneously by exploiting the commonalities and differences between tasks. MTL expects to learn a machine to solve multiple learning tasks simultaneously, which can be regarded as a MOO problem. Establishing a unified MTL paradigm through MOO is an ongoing and significant area of research [258,259].

### Parallel distributed computing

Nature-inspired intelligent computing paradigms have proven to be an effective method for solving NP-hard problems in practical applications due to their efficient parallelism and distributed nature. Given the rapid progress of the information age and the emergence of big data, NP-hard problems grow in scale and complexity. Existing methods are computationally expensive due to the large search space and expensive fitness evaluation [260]. Furthermore, most paradigms can only be iterated serially in computers. Therefore, the parallel distributed nature-inspired intelligent computing paradigms are focused on by researchers.

Many studies have explored parallel and distributed versions of nature-inspired intelligent computing paradigms, which fully exploit its parallelism to reduce computation time [261]. The most intuitive idea is to directly use multiple distributed computing resources to perform parallel operations at the hardware level. In addition, distributed EAs are divided into 2 types at the software level: “distribution of population” and “distribution of dimensional” [249]. The “distribution of population” model distributes individuals in a population across multiple processors or compute nodes, such as the master–slave approach and the island approach, while the “distribution of dimensional” model distributes problem dimensions, such as the agent-based approach and the co-evolutionary approach. Furthermore, there is extensive research on integrating MOO problems with parallel distributed computing [262,263]. Improving the scalability and convergence of parallel nature-inspired intelligent computing paradigms is currently the most difficult challenge.

### Trusted federated learning

Safe and efficient federated learning is an important machine learning paradigm to achieve privacy protection, which can meet the needs of users and market regulation. Trusted federated learning is indispensable in any multi-party AI modeling process, with privacy protection, model performance, and algorithm efficiency at its core. Due to the parallelism and gradient-free, nature-inspired intelligent computing paradigms can enhance fair and trusted federated learning. These paradigms contribute to achieving a general AI paradigm that ensures supervised, controllable efficiency, and interpretable decision-making [264,265].

### Automatic learning

Automated machine learning (AutoML) is an automation technology that enables machine learning models to adaptively solve real-world tasks [266]. Generally, automatic deep learning (ADL) is composed of neural network architecture search (NAS), network parameter optimization, rule learning, and hyperparameter configuration. These problems present challenges such as nonlinearity, nonconvexity, high trial-and-error costs, and combinatorial explosion.

NAS [267] is one of the most popular research areas in AutoML. NAS based on RL is often limited by computational costs and can be unstable. Gradient-based methods often fall into local optima, leading to the discovery of ill-conditioned architectures [87]. Due to their gradient-free, self-adjusting, and self-evolving capabilities, a series of nature-inspired intelligent computing paradigms have been proposed to solve the above problems in recent years [37,268,269]. These techniques can flexibly provide low-cost solutions to complex NAS problems. With the development of general AI, large model-based evolutionary NAS provides a promising direction to further reduce the cost of architecture evaluation [270,271].

For network parameter optimization, gradient-based methods are commonly used. But they are subject to limitations such as “scaling problem” and easy to fall into a massive number of local optima and saddle points [272]. EAs with stochasticity provide theoretical guidance for jumping out of saddle points [273]. In rule learning, nature-inspired intelligent computing paradigms should be further explored in areas such as loss function search, self-evolution learning mechanism, and meta-learning [34]. For the hyperparameter configuration, the impact of each hyperparameter on a deep learning model is interdependent [274]. A set of hyperparameters is critical to the performance of the model, which relies heavily on the researcher’s tuning experience and resources. Simple automated methods based on grid search and random search encounter the effects of dimensional catastrophe. The large-scale EA provides a promising solution for solving hyperparameter optimization [248].

### Robotics based on biological-inspired paradigms

Biological-inspired robots aim to design robots that can dynamically interact with their environment. Such robots tend to help humans work in harsh conditions [275]. A prominent example is the Spot robot dog, which mimics the behavioral movements of a dog [276]. In addition, octopus robots, which are soft-bodied and highly agile, emulate the movements of octopuses [277].

In swarm robotics, multiple robots collaborate as a system to solve real-world problems by interacting with each other and combining their individual actions [278]. Such robots are characterized by flexibility, scalability, robustness, autonomy, self-organization, self-assembly, and decentralization [279]. Biologically inspired swarm robots leverage swarm behaviors observed in nature. Bredeche and Fontbonne [280] introduced social learning-related algorithms for swarm robot deployment. Li et al*.* proposed a mobile robot based on an improved artificial fish swarm algorithm for path planning [281]. In the future, it is also an intriguing direction to combine biological-inspired paradigms with biology to create swarm robots (artificial life) in natural systems.

Robots based on swarm intelligence mechanisms [282] also inspire new technology such as nanorobots in medicine [283], robots in space, and social robotics for specific groups [284]. Beyond hardware limitations, there is still significant potential for research on the safety, reliability, and communication efficiency of swarm robots [285,286].

## Summary and Outlook

Nature-inspired intelligent computing paradigms draw inspiration from the myriad of astounding rules and phenomena observed in nature. Over the years, these paradigms have offered potent solutions to a wide range of practical and complex challenges. This review provides a comprehensive summary of the natural mechanisms underpinning these advanced paradigms, categorizing them into 4 groups: evolutionary-based, biological-based, social-cultural-based, and science-based, each reflecting a unique aspect of the natural world. Additionally, the paper delves into the complex connections between these paradigms and natural mechanisms, revealing the commonalities of nature-inspired intelligent computing paradigms. These commonalities provide a solid algorithmic foundation to avoid designing unreasonable “metaphors”.

Based on a detailed analysis of natural mechanisms, we summarize key challenges facing the nature-inspired intelligent computing paradigm. The development of general AI offers new opportunities to address these challenges. Benefiting from flexible adaptability, nature-inspired intelligent computing paradigms can be combined with advanced large models, such as ChatGPT, for collaborative optimization. Inspired by lessons learned from nature, these synergistic mechanisms have the potential to foster more adaptive and efficient AI systems.

## References

[1] Suzuki R, Arita T. An evolutionary model of personality traits related to cooperative behavior using a large language model. Sci Rep. 2024;14:5989. 38503778 10.1038/s41598-024-55903-y PMC10951268

[2] Bai H, Cheng R, Jin Y. Evolutionary reinforcement learning: A survey. Intell Comput. 2023;2:0025.

[3] Gupta A, Zhou L, Ong YS, Chen Z, Hou Y. Half a dozen real-world applications of evolutionary multitasking, and more. IEEE Comput Intell Mag. 2022;17(2):49–66.

[4] Omidvar MN, Li X, Yao X. A review of population-based metaheuristics for large-scale black-box global optimization—Part I. IEEE Trans Evol Comput. 2022;26(5):802–822.

[5] Ren FJ, Zhou YY, Deng JW, Matsumoto K, Feng D, She TH, Jiao ZY, Liu Z, Li TH, Nakagawa S, et al. Tracking emotions using an evolutionary model of mental state transitions: Introducing a new paradigm. Intell Comput. 2024;3:0075.

[6] Yao X. Evolving artificial neural networks. Proc IEEE. 1999;87:1423–1447.

[7] Telikani A, Tahmassebi A, Banzhaf W, Gandomi AH. Evolutionary machine learning: A survey. ACM Comput Surv. 2021;54(8):1–35.

[8] Zhu S, Yu T, Xu T, Chen H, Dustdar S, Gigan S, Gunduz D, Hossain E, Jin Y, Lin F, et al. Intelligent computing: The latest advances, challenges, and future. Intell Comput. 2023;2:0006.

[9] Kabiri E, Maftouni N. Multiple objective energy optimization of a trade center building based on genetic algorithm using ecological materials. Sci Rep. 2024;14(1):9366. 38653981 10.1038/s41598-024-58515-8 PMC11039617

[10] Martinho AD, Hippert HS, Goliatt L. Short-term streamflow modeling using data-intelligence evolutionary machine learning models. Sci Rep. 2023;13:13824. 37620432 10.1038/s41598-023-41113-5 PMC10449879

[11] Bilal A, Imran A, Baig TI, Liu X, Abouel Nasr E, Long H. Breast cancer diagnosis using support vector machine optimized by improved quantum inspired grey wolf optimization. Sci Rep. 2024;14:10714. 38730250 10.1038/s41598-024-61322-w PMC11087531

[12] Lin L, Guo J, Liu L. Multi-scene application of intelligent inspection robot based on computer vision in power plant. Sci Rep. 2024;14:10657. 38724514 10.1038/s41598-024-56795-8 PMC11522562

[13] Miikkulainen R, Forrest S. A biological perspective on evolutionary computation. Nat Mach Intell. 2021;3:9–15.

[14] Chelly Dagdia Z, Avdeyev P, Bayzid M. Biological computation and computational biology: Survey, challenges, and discussion. Artif Intell Rev. 2021;54:4169–4235.

[15] Kumar SR and Singh KD. Nature-inspired optimization algorithms: research direction and survey. arXiv. 2021. 10.48550/arXiv.2102.04013

[16] Sachan RK, Kushwaha DS. Inspirations from nature for meta-heuristic algorithms: A survey. Recent Adv Comput Sci Commun. 2021;14(6):1706–1718.

[17] Tang J, Liu G, Pan Q. A review on representative swarm intelligence algorithms for solving optimization problems: Applications and trends. IEEE/CAA J Autom Sin. 2021;8(10):1627–1643.

[18] Torres-Treviño L. A 2020 taxonomy of algorithms inspired on living beings behavior. arXiv. 2021. 10.48550/arXiv.2106.04775

[19] Eiben AE, Smith J. From evolutionary computation to the evolution of things. Nature. 2015;521:476–482. 26017447 10.1038/nature14544

[20] Ali AF, Mostafa A, Sayed GI, Elfattah MA, Hassanien AE. Nature inspired optimization algorithms for CT liver segmentation. In: Dey N, Bhateja V, Hassanien A, editors. *Medical imaging in clinical applications: algorithmic and computer-based approaches*. Cham: Springer; 2016. p. 431–460.

[21] Houssein EH, Ed H, Oliva D, Elngar AA, Shaban H. Multi-level thresholding image segmentation based on nature-inspired optimization algorithms: A comprehensive review. In: Oliva D, Houssein EH, Hinojosa S, editors. *Metaheuristics in machine learning: Theory and applications*. Cham: Springer; 2021. p. 239–265.

[22] Khamparia A, Khanna A, Nguyen NG, Nguyen BL. *Nature-inspired optimization algorithms: Recent advances in natural computing and biomedical applications*. Berlin, Boston: De Gruyter; 2021.

[23] Jin Y, Wang H, Sun C. *Data-driven evolutionary optimization*. Cham: Springer; 2021.

[24] Del Ser J, Osaba E, Molina D, Yang X-S, Salcedo-Sanz S, Camacho D, Das S, Suganthan PN, Coello Coello CA, Herrera F. Bio-inspired computation: Where we stand and what’s next. Swarm Evol Comput. 2019;48:220–250.

[25] Yang XS. Nature-inspired optimization algorithms: Challenges and open problems. J Comput Sci. 2020;46:101104.

[26] Dalavi AM, Gomes A, Husain AJ. Bibliometric analysis of nature inspired optimization techniques. Comput Ind Eng. 2022;169:108161.

[27] Omidvar MN, Li X, Yao X. A review of population-based metaheuristics for large-scale black-box global optimization—Part II. IEEE Trans Evol Comput. 2022;26(5):823–843.

[28] Kudela J. A critical problem in benchmarking and analysis of evolutionary computation methods. Nat Mach Intell. 2022;4:1238–1245.

[29] Gharehchopogh FS. Quantum-inspired metaheuristic algorithms: Comprehensive survey and classification. Artif Intell Rev. 2023;56:5479–5543.

[30] Piotrowski AP, Napiorkowski JJ. Some metaheuristics should be simplified. Inf Sci. 2018;427:32–62.

[31] Campelo F, Aranha C. Lessons from the evolutionary computation bestiary. Artif Life. 2023;29(4):421–432. 37432094 10.1162/artl_a_00402

[32] de Armas J, Lalla-Ruiz E, Tilahun SL, Voß S. Similarity in metaheuristics: A gentleste towards a comparison methodology. Nat Comput. 2022;21:265–287.

[33] Swan J, Adriaensen S, Brownlee AE, Hammond K, Johnson CG, Kheiri A, Krawiec F, Merelo JJ, Minku LL, Özcan E, et al. Metaheuristics “in the large”. Eur J Oper Res. 2022;297(2):393–406.

[34] Li N, Ma L, Yu G, Xue B, Zhang M, Jin Y. Survey on evolutionary deep learning: Principles, algorithms, applications, and open issues. ACM Comput Surv. 2023;56(2):1–34.

[35] Pei W, Xue B, Zhang M, Shang L, Yao X, Zhang Q. A survey on unbalanced classification: How can evolutionary computation help? IEEE Trans Evol Comput. 2024;28(2):353–373.

[36] Nssibi M, Manita G, Korbaa O. Advances in nature-inspired metaheuristic optimization for feature selection problem: A comprehensive survey. Comput Sci Rev. 2023;49:100559.

[37] Zhao J, Jiao L, Wang C, Liu X, Liu F, Li L, Ma M, Yang S. Knowledge guided evolutionary transformer for remote sensing scene classification. IEEE Trans Circuits Syst Video Technol. 2024;1–16.

[38] Darwin C. *The origin of species*. New York: P. F. Collier; 1909.

[39] Back T. *Evolutionary algorithms in theory and practice: Evolution strategies, evolutionary programming, genetic algorithms.* Oxford (UK): Oxford Univ. Press; 1996.

[40] Lehman J, Clune J, Misevic D, Adami C, Altenberg L, Beaulieu J, Bentley PJ, Bernard S, Beslon G, Bryson DM, et al. The surprising creativity of digital evolution: A collection of anecdotes from the evolutionary computation and artificial life research communities. Artif Life. 2020;26(2):274–306. 32271631 10.1162/artl_a_00319

[41] Fogel DB, Hays TJ, Hahn SL, Quon J. A self-learning evolutionary chess program. Proc IEEE. 2004;92(12):1947–1954.

[42] Young AI, Benonisdottir S, Przeworski M, Kong A. Deconstructing the sources of genotypephenotype associations in humans. Science. 2019;365(6460):1396–1400. 31604265 10.1126/science.aax3710 PMC6894903

[43] Benfey PN, Mitchell-Olds T. From genotype to phenotype: Systems biology meets natural variation. Science. 2008;320(5875):495–497. 18436781 10.1126/science.1153716 PMC2727942

[44] Lind MI, Spagopoulou F. Evolutionary consequences of epigenetic inheritance. Heredity. 2018;121:205–209. 29976958 10.1038/s41437-018-0113-y PMC6082883

[45] McBride PD, Gillman LN, Wright SD. Current debates on the origin of species. J Biol Educ. 2009;43(3):104–107.

[46] Poelwijk FJ, Socolich M, Ranganathan R. Learning the pattern of epistasis linking genotype and phenotype in a protein. Nat Commun. 2019;10:4213. 31527666 10.1038/s41467-019-12130-8 PMC6746860

[47] Yang D, Jin Y, He X, Dong A, Wang J, Wu R. Inferring multilayer interactome networks shaping phenotypic plasticity and evolution. Nat Commun. 2021;12:5304. 34489412 10.1038/s41467-021-25086-5 PMC8421358

[48] Wu D, Arakawa H, Fujita A, Hashimoto H, Hibi M, Naruse K, Kamei Y, Sato C, Kitajima K. A point-mutation in the C-domain of CMP-sialic acid synthetase leads to lethality of medaka due to protein insolubility. Sci Rep. 2021;11:23211. 34853329 10.1038/s41598-021-01715-3 PMC8636478

[49] Rodgers K, McVey M. Error-prone repair of DNA double-strand breaks. J Cell Physiol. 2016;231:15–24. 26033759 10.1002/jcp.25053 PMC4586358

[50] Bawden FC. Effect of nitrous acid on tobacco mosaic virus: Mutation or selection? Nature. 1959;184:BA27–BA29. 13848063

[51] Shi J, He J, Lin J, Sun X, Sun F, Ou C, Jiang C. Distinct response of the hepatic transcriptome to aflatoxin B1 induced hepatocellular carcinogenesis and resistance in rats. Sci Rep. 2016;6:31898. 27545718 10.1038/srep31898 PMC4992951

[52] Ni K, Lan G, Guo N, Culbert A, Luo T, Wu T, Weichselbaum RR, Lin W. Nanoscale metal-organic frameworks for x-ray activated in situ cancer vaccination. Sci Adv. 2020;6(40):eabb5223. 33008911 10.1126/sciadv.abb5223 PMC7852401

[53] Noonan FP, Zaidi MR, Wolnicka-Glubisz A, Anver MR, Bahn J, Wielgus A, Cadet J, Douki T, Mouret S, Tucker MA, et al. Melanoma induction by ultraviolet A but not ultraviolet B radiation requires melanin pigment. Nat Commun. 2012;3:884. 22673911 10.1038/ncomms1893 PMC3621412

[54] Saito A, Irie T, Suzuki R, Maemura T, Nasser H, Uriu K, Kosugi Y, Shirakawa K, Sadamasu K, Kimura I, et al. Enhanced fusogenicity and pathogenicity of SARS-CoV-2 Delta P681R mutation. Nature. 2022;602:300–306. 34823256 10.1038/s41586-021-04266-9 PMC8828475

[55] Crow JF. The high spontaneous mutation rate: Is it a health risk? Proc Natl Acad Sci U.S.A. 1997;94(16):8380–8386. 9237985 10.1073/pnas.94.16.8380 PMC33757

[56] Mendiratta G, Ke E, Aziz M, Liarakos D, Tong M, Stites EC. Cancer gene mutation frequencies for the US population. Nat Commun. 2021;12:5961. 34645806 10.1038/s41467-021-26213-y PMC8514428

[57] Wienert B, Funnell AP, Norton LJ, Pearson RCM, Wilkinson-White LE, Lester K, Vadolas J, Porteus MH, Matthews JM, Quinlan KGR, et al. Editing the genome to introduce a beneficial naturally occurring mutation associated with increased fetal globin. Nat Commun. 2015;6:7085. 25971621 10.1038/ncomms8085

[58] Shahin T, Kuehn HS, Shoeb MR, Gawriyski L, Giuliani S, Repiscak P, Hoeger B, Yüce Petronczki Ö, Bal SK, Zoghi S, et al. Germline biallelic mutation affecting the transcription factor Helios causes pleiotropic defects of immunity. Sci Immunol. 2021;6(65):eabe3981. 34826259 10.1126/sciimmunol.abe3981 PMC7612971

[59] Sawyer SA, Parsch J, Zhang Z, Hartl DL. Prevalence of positive selection among nearly neutral amino acid replacements in Drosophila. Proc Natl Acad Sci U S A. 2007;104:6504–6510. 17409186 10.1073/pnas.0701572104 PMC1871816

[60] Smerlak M. Neutral quasispecies evolution and the maximal entropy random walk. Sci Adv. 2021;7(16):eabb2376. 33853768 10.1126/sciadv.abb2376 PMC8046360

[61] Lande R. Fisherian and Wrightian theories of speciation. Genome. 1989;31(1):221–227. 2687093 10.1139/g89-037

[62] Ambjørn SM, Duxin JP, Hertz E, Nasa I, Duro J, Kruse T, Lopez-Mendez B, Rymarczyk B, Cressey LE, van Overeem Hansen T, et al. A complex of BRCA2 and PP2A-B56 is required for DNA repair by homologous recombination. Nat Commun. 2021;12:5748. 34593815 10.1038/s41467-021-26079-0 PMC8484605

[63] Rivera MC, Lake JA. The ring of life provides evidence for a genome fusion origin of eukaryotes. Nature. 2004;431:152–155. 15356622 10.1038/nature02848

[64] Mayr E. The objects of selection. Proc Natl Acad Sci U S A. 1997;94(6):2091–2094. 9122151 10.1073/pnas.94.6.2091 PMC33654

[65] Okada K, Katsuki M, Sharma MD, Kiyose K, Seko T, Okada Y, Wilson AJ, Hosken DJ. Natural selection increases female fitness by reversing the exaggeration of a male sexually selected trait. Nat Commun. 2021;12:3420. 34103535 10.1038/s41467-021-23804-7 PMC8187464

[66] Whigham PA, Dick G, Maclaurin J. On the mapping of genotype to phenotype in evolutionary algorithms. Genet Program Evolvable Mach. 2017;18:353–361. 10.1007/s10710-017-9292-1 PMC5618731 29033669

[67] Sampson JR. Adaptation in natural and artificial systems (John H. Holland). Society for Industrial and Applied Mathematics. 1976.

[68] Holland JH. *Adaptation in natural and artificial systems: An introductory analysis with applications to biology, control, and artificial intelligence.* Cambridge (MA): MIT Press; 1992.

[69] Back T. Selective pressure in evolutionary algorithms: a characterization of selection mechanisms. Paper presented at: Proceedings of the First IEEE Conference on Evolutionary Computation. IEEE World Congress on Computational Intelligence; 1994; Orlando, FL, USA.

[70] Deb K, Agrawal RB. Simulated binary crossover for continuous search space. Complex Syst. 1995;9:115–148.

[71] Deb K, Goyal M. A combined genetic adaptive search (GeneAS) for engineering design. Comput Sci Inform. 1996;26(4):30–45.

[72] Krasnogor N, Smith J. A tutorial for competent memetic algorithms: Model, taxonomy, and design issues. IEEE Trans Evol Comput. 2005;9:474–488.

[73] Li X, Wu K, Zhang X, Wang H, Liu J. B2Opt: Learning to optimize black-box optimization with little budget. arXiv. 2023. https://doi.org/10.48550/arXiv.2304.11787.

[74] Lange R, Schaul T, Chen Y, Zahavy T, Dallibard V, Lu C, Singh S, Flennerhag S. Discovering evolution strategies via meta-black-box optimization. Paper presented at: Proceedings of the Companion Conference on Genetic and Evolutionary Computation; 2023; New York, NY, USA.

[75] Lange RT, Tian Y, Tang Y. Evolution Transformer: In-context evolutionary optimization. arXiv. 2024. 10.48550/arXiv.2403.02985

[76] Rechenberg I. *Evolutionsstrategie: Optimierung technischer systeme nach prinzipien der biologischen evolution*. Stuttgart: Frommann–Holzboog; 1973.

[77] Hansen N. The CMA evolution strategy: A tutorial. arXiv. 2016. https://doi.org/10.48550/arXiv.1604.00772.

[78] Glasmachers T, Krause O. Convergence analysis of the hessian estimation evolution strategy. Evol Comput. 2022;30(1):27–50. 34779840 10.1162/evco_a_00295

[79] Deng K, Hu J. Decentralized projected Riemannian gradient method for smooth optimization on compact submanifolds. arXiv. 2023. https://doi.org/10.48550/arXiv.2304.08241

[80] Hu J, Ao R, So AMC, Yang M, Wen Z. Riemannian natural gradient methods. SIAM J Sci Comput. 2024;46:A204–A231.

[81] Glasmachers T, Krause O. The Hessian estimation evolution strategy. In: *International Conference on Parallel Problem Solving from Nature (PPSN XVI)*. Cham: Springer; 2020. p. 597–609.

[82] Fogel DB. *Artificial intelligence through simulated evolution*. Chichester: Wiley-IEEE Press; 1998.

[83] Koza JR. Evolution of subsumption using genetic programming. In: *Proceedings of the First European Conference on Artificial Life*. Cambridge (MA): MIT Press; 1992. p. 110–119.

[84] Lehman J, Gordon J, Jain S, Ndousse K, Yeh C, Stanley KO. Evolution through large models. In: *Handbook of evolutionary machine learning*. Singapore: Springer; 2023. p. 331–366.

[85] Tanneberg D, Rueckert E, Peters J. Evolutionary training and abstraction yields algorithmic generalization of neural computers. Nat Mach Intell. 2020;2(12):753–763.

[86] Zhou X, Qin A, Gong M, Tan KC. A survey on evolutionary construction of deep neural networks. IEEE Trans Evol Comput. 2021;25(5):894–912.

[87] Liu Y, Sun Y, Xue B, Zhang M, Yen GG, Tan KC. A survey on evolutionary neural architecture search. IEEE Trans Neur Netw Learn Syst. 2021;34(2):550–570. 10.1109/TNNLS.2021.3100554 34357870

[88] OpenAI; Achiam J, Adler S, Agarwal S, Ahmad L, Akkaya I, Aleman FL, Almeida D, Altenschmidt J, Altman S, et al. Gpt-4 technical report. arXiv. 2023. 10.48550/arXiv.2303.08774

[89] Chao W, Zhao J, Jiao L, Li L, Liu F, Yang S. A match made in consistency heaven: when large language models meet evolutionary algorithms. arXiv. 2024. 10.48550/arXiv.2401.10510

[90] Yao Y, Liu F, Cheng J, Zhang Q. Evolve cost-aware acquisition functions using large language models. arXiv. 2024. 10.48550/arXiv.2404.16906 41505501

[91] Romera-Paredes B, Barekatain M, Novikov A, Balog M, Kumar MP, Dupont E, Ruiz FJR, Ellenberg JS, Wang P, Fawzi O, et al. Mathematical discoveries from program search with large language models. Nature. 2024;625(7995):468–475. 38096900 10.1038/s41586-023-06924-6 PMC10794145

[92] Huang S, Yang K, Qi S, Wang R. When large language model meets optimization. arXiv. 2024. 10.48550/arXiv.2405.10098

[93] Dressler F, Akan OB. Bio-inspired networking: From theory to practice. IEEE Commun Mag. 2010;48:176–183.

[94] Liu Y and Passino KM. *Swarm intelligence: Literature overview*. Columbus (OH): The Ohio State University; 2000.

[95] Abrahms B, Teitelbaum CS, Mueller T, Converse SJ. Ontogenetic shifts from social to experiential learning drive avian migration timing. Nat Commun. 2021;12(1):7326. 34916500 10.1038/s41467-021-27626-5 PMC8677782

[96] Pennisi E. Social animals prove their smarts. Science. 2006;312(5781):1734–1738. 16794055 10.1126/science.312.5781.1734

[97] Van de Waal E, Borgeaud C, Whiten A. Potent social learning and conformity shape a wild primate’s foraging decisions. Science. 2013;340(6131):483–485. 23620053 10.1126/science.1232769

[98] Sliwa J. Toward collective animal neuroscience. Science. 2021;374(6566):397–398. 34672744 10.1126/science.abm3060

[99] Beni G, Wang J. Swarm intelligence in cellular robotic systems. In: Robots and biological systems: towards a new bionics? Heidelberg: Springer; 1993. p. 703–712.

[100] Bonabeau E, Theraulaz G, Dorigo M. *Swarm intelligence: From natural to artificial systems*. Oxford (UK): Oxford Univ. Press; 1999.

[101] Nedjah N, Mourelle LDM, Morais RG. Inspiration-wise swarm intelligence meta-heuristics for continuous optimisation: A survey-part I. Int J Bio-Insp Comput. 2020;15(4):207–223.

[102] Choe JC. *Encyclopedia of animal behavior.* San Diego: Academic Press; 2019.

[103] Clark CW, Mangel M. The evolutionary advantages of group foraging. Theor Popul Biol. 1986;30:45–75.

[104] Giraldeau LA, Caraco T. Social foraging theory. Princeton (NJ): Princeton Univ. Press; 2018.

[105] Turrin C, Fagan NA, Dal Monte O, Chang SW. Social resource foraging is guided by the principles of the marginal value theorem. Sci Rep. 2017;7(1):1–13. 28900299 10.1038/s41598-017-11763-3 PMC5596022

[106] Charnov EL. Optimal foraging, the marginal value theorem. Theor Popul Biol. 1976;9(2):129–136. 1273796 10.1016/0040-5809(76)90040-x

[107] Kennedy J, Eberhart R. Particle swarm optimization. Paper presented at: *Proceedings of ICNN’95 International Conference on Neural Networks*; 1995; Perth, WA, Australia.

[108] Dorigo M, Birattari M, Stutzle T. Ant colony optimization. IEEE Comput Intell Mag. 2006;1:28–39.

[109] Karaboga D. An idea based on honey bee swarm for numerical optimization. Technical report-tr06. Erciyes University; 2005. p. 1–10.

[110] Mirjalili S, Mirjalili SM, Lewis A. Grey wolf optimizer. Adv Eng Softw. 2014;69:46–61.

[111] Poli R, Kennedy J, Blackwell T. Particle swarm optimization. Swarm Intell. 2007;1:33–57.

[112] Hansson LA, Akesson S. Animal movement across scales. Oxford (UK): Oxford Univ. Press; 2014.

[113] Darwin C. Origin of certain instincts. Nature. 1873;7(179):417–418.

[114] Mirjalili S. Moth-flame optimization algorithm: A novel nature-inspired heuristic paradigm. Knowl-Based Syst. 2015;89:228–249.

[115] Yang XS, He X. Bat algorithm: Literature review and applications. Int J Bio-Insp Comput. 2013;5:141–149.

[116] Dhiman G, Singh KK, Slowik A, Chang V, Yildiz AR, Kaur A, Garg M. EMoSOA: A new evolutionary multi-objective seagull optimization algorithm for global optimization. Int J Mach Learn Cybern. 2021;12(7):571–596.

[117] Macdonald S, Mason C. Predation of migrant birds by gulls. British Birds. 1973;66:361–363.

[118] Dhiman G, Kumar V. Seagull optimization algorithm: Theory and its applications for large-scale industrial engineering problems. Knowl-Based Syst. 2019;165:169–196.

[119] Wang GG, Deb S, Cui Z. Monarch butterfly optimization. Neural Comput Applic. 2019;31:1995–2014.

[120] Bookman MA. Sensitivity of the homing pigeon to an earth-strength magnetic field. In: Animal migration, navigation, and homing. Berlin: Springer; 1978. p. 127–134.

[121] Duan H, Qiao P. Pigeon-inspired optimization: A new swarm intelligence optimizer for air robot path planning. Int J Intell Comput Cybern. 2014;7:24–37.

[122] Ayala FJ, Hubbell SP, Avise JC. *In the light of evolution: Volume II: Biodiversity and extinction. Vol. 2*. Washington (DC): National Academies Press; 2009. 25009919

[123] Cox FE. History of human parasitology. Clin Microbiol Rev. 2002;15(4):595–612. 12364371 10.1128/CMR.15.4.595-612.2002 PMC126866

[124] Rabajante JF, Tubay JM, Ito H, Uehara T, Kakishima S, Morita S, Yoshimura J, Ebert D. Host-parasite red queen dynamics with phase-locked rare genotypes. Sci Adv. 2016;2(3):e1501548. 26973878 10.1126/sciadv.1501548 PMC4783124

[125] Medina I, Kilner RM, Langmore NE. From micro-to macroevolution: Brood parasitism as a driver of phenotypic diversity in birds. Curr Zool. 2020;66(5):515–526. 33293930 10.1093/cz/zoaa033 PMC7705515

[126] Shehab M, Khader AT, Al-Betar MA. A survey on applications and variants of the cuckoo search algorithm. Appl Soft Comput. 2017;61:1041–1059.

[127] Yang XS, Deb S. Cuckoo search via Lévy flights. Paper presented at: 2009 World Congress on Nature & Biologically Inspired Computing (NaBIC); 2009; Coimbatore, India.

[128] Mishra S, Nayak PC, Prusty UC, Prusty RC. Implementation of a hybrid cuckoo search and pattern search algorithm for frequency control of the microgrid system. Paper presented at: 2020 International Conference on Renewable Energy Integration into Smart Grids: A Multidisciplinary Approach to Technology Modelling and Simulation (ICREISG); 2020; Bhubaneswar, India.

[129] Ouaarab A, Ahiod B, Yang XS. Discrete cuckoo search algorithm for the travelling salesman problem. Neural Comput Appl. 2014;24(7-8):1659–1669.

[130] Gandomi AH, Yang XS, Alavi AH. Cuckoo search algorithm: A metaheuristic approach to solve structural optimization problems. Eng Comput. 2013;29(1):17–35.

[131] Bomze IM. Lotka-Volterra equation and replicator dynamics: A two-dimensional classification. Biol Cybern. 1983;48(3):201–211.

[132] Mehrabian AR, Lucas C. A novel numerical optimization algorithm inspired from weed colonization. Eco Inform. 2006;1(4):355–366.

[133] Sedighy S, Mallahzadeh A, Soleimani M, Rashed-Mohassel J. Optimization of printed Yagi antenna using invasive weed optimization (IWO). IEEE Antenn Wirel Propag Lett. 2010;9:1275–1278.

[134] Rad HS, Lucas C. A recommender system based on invasive weed optimization algorithm. Paper presented at: 2007 IEEE Congress on Evolutionary Computation; 2007; Singapore.

[135] Darwin C. The descent of man, and selection in relation to sex. Princeton (NJ): Princeton Univ. Press; 2008.

[136] Borgia G. Bower destruction and sexual competition in the satin bowerbird (Ptilonorhynchus violaceus). Behav Ecol Sociobiol. 1985;18:91–100.

[137] Moosavi SHS, Bardsiri VK. Satin bowerbird optimizer: A new optimization algorithm to optimize ANFIS for software development effort estimation. Eng Appl Artif Intell. 2017;60:1–15.

[138] Zervoudakis K, Tsafarakis S. A mayfly optimization algorithm. Comput Ind Eng. 2020;145:106559.

[139] Hayyolalam V, Kazem AAP. Black widow optimization algorithm: A novel meta-heuristic approach for solving engineering optimization problems. Eng Appl Artif Intell. 2020;87:103249.

[140] Pierezan J and Dos Santos Coelho L. Coyote optimization algorithm: A new metaheuristic for global optimization problems. Paper presented at: 2018 IEEE Congress on Evolutionary Computation (CEC); 2018; Rio de Janeiro, Brazil.

[141] Wang GG, Deb S, Coelho LdS. Elephant herding optimization. Paper presented at: 2015 3rd International Symposium on Computational and Business Intelligence (ISCBI); 2015; Bali, Indonesia.

[142] Chu SC, Tsai PW, Pan JS. Cat swarm optimization. In: *Pacific Rim international conference on artificial intelligence*. 2006:854–8.

[143] Yang XS, Deb S. Eagle strategy using Lévy walk and firefly algorithms for stochastic optimization. In: *Nature inspired cooperative strategies for optimization (NICSO 2010)*. Berlin: Springer; 2010. p. 101–111.

[144] Paton R, Bolouri H, Holcombe WML, Parish JH, Tateson R. *Computation in cells and tissues: Perspectives and tools of thought*. Heidelberg: Springer Science & Business Media; 2013.

[145] Turvey SE, Broide DH. Innate immunity. J Allergy Clin Immunol. 2010;125:S24–S32. 19932920 10.1016/j.jaci.2009.07.016 PMC2832725

[146] Lewis DE, Blutt SE. 2 - Organization of the immune system. In: Rich RR, Fleisher TA, Shearer WT, Schroeder HW, Frew AJ, Weyand CM. *Clinical immunology (fifth edition)*. London: Elsevier; 2019. p. 19–38.e1.

[147] Martin TD, Patel RS, Cook DR, Choi MY, Patil A, Liang AC, Li MZ, Haigis KM, Elledge SJ. The adaptive immune system is a major driver of selection for tumor suppressor gene inactivation. Science. 2021;373(6561):1327–1335. 34529489 10.1126/science.abg5784 PMC13397623

[148] Parija SC. *Textbook of microbiology and immunology.* Singapore: Springer; 2023.

[149] Dasgupta D, Nino F. Immunological computation: Theory and *applications*. New York: Auerbach Publications ; 2008.

[150] Hofmeyr SA, Forrest S. Architecture for an artificial immune system. Evol Comput. 2000;8:443–473. 11130924 10.1162/106365600568257

[151] Cohn M, Av Mitchison N, Paul WE, Silverstein AM, Talmage DW, Weigert M. Reflections on the clonal-selection theory. Nat Rev Immunol. 2007;7(10):823–830. 17893695 10.1038/nri2177

[152] Murugan R, Buchauer L, Triller G, Kreschel C, Costa G, Pidelaserra Martí G, Imkeller K, Busse CE, Chakravarty S, Sim BKL, et al. Clonal selection drives protective memory B cell responses in controlled human malaria infection. Sci Immunol. 2018;3(20):eaap8029. 29453292 10.1126/sciimmunol.aap8029

[153] Kurosaki T, Kometani K, Ise W. Memory B cells. Nat Rev Immunol. 2015;15:149–159. 25677494 10.1038/nri3802

[154] De Castro LN, Von Zuben FJ. Learning and optimization using the clonal selection principle. IEEE Trans Evol Comput. 2002;6(3):239–251.

[155] Anaya JM, Shoenfeld Y, Rojas-Villarraga A, Levy R, Cervera R. *Autoimmunity from bench to bedside*. Bogota: EI Rosario University Press; 2013. 29087650

[156] Timmis J, Neal M. A resource limited artificial immune system for data analysis. Knowl Based Syst. 2001;14(3):121–130.

[157] Nunes de Casto L, Von Zuben F. An evolutionary immune network for data clustering. Paper presented at: Proceedings. Vol.1. Sixth Brazilian Symposium on Neural Networks; 2000; Rio de Janeiro, Brazil.

[158] Von Boehmer H, Kisielow P. Self-nonself discrimination by T cells. Science. 1990;248(4961):1369–1373. 1972594 10.1126/science.1972594

[159] Forrest S, Perelson A, Allen L, Cherukuri R. Self-nonself discrimination in a computer. Paper presented at: Proceedings of 1994 IEEE Computer Society Symposium on Research in Security and Privacy; 1994; Oakland, CA, USA.

[160] The MP, Model D. A renewed sense of self. Science. 2002;296(1):301–305. 11951032 10.1126/science.1071059

[161] Aickelin U, Greensmith J. Sensing danger: Innate immunology for intrusion detection. Inf Secur Tech Rep. 2007;12(4):218–227.

[162] Ledford H. What the immune response to the coronavirus says about the prospects for a vaccine. Nature. 2020;585(7823):20–21. 32811981 10.1038/d41586-020-02400-7

[163] Jiao L, Wang L. A novel genetic algorithm based on immunity. IEEE Trans Syst Man Cybern Syst Hum. 2000;30:552–561.

[164] Shi Y. Brain storm optimization algorithm. Paper presented at: International Conference in Swarm Intelligence; 2011; Chongqing, China.

[165] Cheng S, Qin Q, Chen J, Shi Y. Brain storm optimization algorithm: A review. Artif Intell Rev. 2016;46:445–458.

[166] Shen L. Research and application of v-SVR based on brain storm optimization algorithm [thesis]. Lanzhou University; 2014.

[167] Reynolds RG. An introduction to cultural algorithms. In: *Proceedings of the Third Annual Conference on Evolutionary Programming*. River Edge: World Scientific; 1994. p. 131–139.

[168] Maheri A, Jalili S, Hosseinzadeh Y, Khani R, Miryahyavi M. A comprehensive survey on cultural algorithms. Swarm Evol Comput. 2021;62(1):100846.

[169] Atashpaz-Gargari E, Lucas C. Imperialist competitive algorithm: An algorithm for optimization inspired by imperialistic competition. Paper presented at: 2007 IEEE Congress on Evolutionary Computation; 2007; Singapore.

[170] Hosseini S, Al KA. A survey on the imperialist competitive algorithm metaheuristic: Implementation in engineering domain and directions for future research. Appl Soft Comput. 2014;24:1078–1094.

[171] Behnamian J, Zandieh M. A discrete colonial competitive algorithm for hybrid flowshop scheduling to minimize earliness and quadratic tardiness penalties. Expert Syst Appl. 2011;38(12):14490–14498.

[172] Rao R, Savsani V, Vakharia D. Teaching–learning-based optimization: A novel method for constrained mechanical design optimization problems. Comput Aided Des. 2011;43(3):303–315.

[173] Zou F, Chen D, Xu Q. A survey of teaching–learning-based optimization. Neurocomputing. 2019;335(7-9):366–383.

[174] Ma Y, Zhang X, Song J, Chen L. A modified teaching–learning-based optimization algorithm for solving optimization problem. Knowl-Based Syst. 2021;212(7):106599.

[175] Ab M, Yc C, Yin H, Sz C. Crisscross optimization algorithm and its application. Knowl-Based Syst. 2014;67:218–229.

[176] Punnathanam V, Kotecha P. Yin-yang-pair optimization: A novel lightweight optimization algorithm. Eng Appl Artif Intell. 2016;54:62–79.

[177] Askari Q, Saeed M, Younas I. Heap-based optimizer inspired by corporate rank hierarchy for global optimization. Expert Syst Appl. 2020;161:113702.

[178] Satapathy S, Naik A. Social group optimization (SGO): A new population evolutionary optimization technique. Complex Intell Syst. 2016;2(3):173–203.

[179] Van Laarhoven PJ, Aarts EH. Simulated annealing. In: *Simulated annealing: Theory and applications*. Dordrecht: Springer;1987. p. 7–15.

[180] Bandyopadhyay R, Basu A, Cuevas E, Sarkar R. Harris Hawks optimisation with simulated annealing as a deep feature selection method for screening of COVID-19 CT-scans. Appl Soft Comput. 2021;111(13):107698. 34276262 10.1016/j.asoc.2021.107698 PMC8277546

[181] Lin SW, Cheng CY, Pourhejazy P, Ying KC. Multi-temperature simulated annealing for optimizing mixed-blocking permutation flowshop scheduling problems. Expert Syst Appl. 2021;165:113837.

[182] Fahimi Z, Mahmoodi M, Nili H, Polishchuk V, Strukov D. Combinatorial optimization by weight annealing in memristive hopfield networks. Sci Rep. 2021;11(1):16383. 34385475 10.1038/s41598-020-78944-5 PMC8361025

[183] Zamfirache IA, Precup RE, Roman RC, Petriu EM. Reinforcement learning-based control using Q-learning and gravitational search algorithm with experimental validation on a nonlinear servo system. Inf Sci. 2022;583(2):99–120.

[184] Rashedi E, Nezamabadi-pour H, Saryazdi S, Saryazdi S. GSA: A gravitational search algorithm. Inf Sci. 2009;179(13):2232–2248.

[185] Rashedi E, Rashedi E, Nezamabadi-pour H. A comprehensive survey on gravitational search algorithm. Swarm Evol Comput. 2018;41(4):141–158.

[186] Han KH, Kim JH. Genetic quantum algorithm and its application to combinatorial optimization problem. Paper presented at: Proceedings of the 2000 Congress on Evolutionary Computation. CEC00 (Cat. No.00TH8512); 2000; La Jolla, CA, USA.

[187] Xiong H, Wu Z, Fan H, Li G, Jiang G. Quantum rotation gate in quantum-inspired evolutionary algorithm: A review, analysis and comparison study. Swarm Evol Comput. 2018;42:43–57.

[188] Yang S, Wang M, Jiao L. A quantum particle swarm optimization. Paper presented at: Proceedings of the 2004 Congress on Evolutionary Computation (IEEE Cat. No.04TH8753); 2004; Portland, OR, USA.

[189] Jiao L, Li Y, Gong M, Zhang X. Quantum-inspired immune clonal algorithm for global optimization. IEEE Trans Syst Man Cybern B Cybern. 2008;38(5):1234–1253. 18784009 10.1109/TSMCB.2008.927271

[190] Hibat-Allah M, Inack EM, Wiersema R, Melko RG, Carrasquilla J. Variational neural annealing. Nat Mach Intell. 2021;3(11):952–961.

[191] Chai Y, Han YJ, Wu YC, Li Y, Dou M, Guo GP. Shortcuts to the quantum approximate optimization algorithm. Phys Rev A. 2022;105(4):042415.

[192] Khoury J, Ovrut BA, Seiberg N, Steinhardt PJ, Turok N. From big crunch to big bang. Phys Rev D. 2002;65(8):086007.

[193] Mirjalili S, Mirjalili SM, Hatamlou A. Multi-verse optimizer: A nature-inspired algorithm for global optimization. Neural Comput Applic. 2016;27(2):495–513.

[194] Kumar KA, Boda R. A multi-objective randomly updated beetle swarm and multi verse optimization for brain tumor segmentation and classification. Comput J. 2021;65(4):171.

[195] Shareef H, Ibrahim AA, Mutlag AH. Lightning search algorithm. Appl Soft Comput. 2015;36(2015):315–333.

[196] Bhandari AK, Singh N, Kumar IV. Lightning search algorithm-based contextually fused multilevel image segmentation. Appl Soft Comput. 2020;91(2):106243.

[197] Birbil SI and Fang SC. An electromagnetism-like mechanism for global optimization. J Glob Optim. 2003;25(3):263–282.

[198] Naderi B, Tavakkoli-Moghaddam R, Khalili M. Electromagnetism-like mechanism and simulated annealing algorithms for flowshop scheduling problems minimizing the total weighted tardiness and makespan. Knowl-Based Syst. 2010;23(2):77–85.

[199] Nematollahi AF, Rahiminejad A, Vahidi B. A novel physical based meta-heuristic optimization method known as lightning attachment procedure optimization. Appl Soft Comput. 2017;59:596–621.

[200] Mohamed M, Youssef AR, Kamel S, Ebeed M. Lightning attachment procedure optimization algorithm for nonlinear non-convex short-term hydrothermal generation scheduling. Soft Comput. 2020;24(21):16225–16248.

[201] Sun S, Ye Z, Yan L, Su J, Wang R. Wrapper feature selection based on lightning attachment procedure optimization and support vector machine for intrusion detection. Paper presented at: 2018 IEEE 4th International Symposium on Wireless Systems within the International Conferences on Intelligent Data Acquisition and Advanced Computing Systems (IDAACSSWS); 2018; Lviv, Ukraine.

[202] Hashim FA, Hussain K, Houssein EH, Mabrouk MS, Al-Atabany W. Archimedes optimization algorithm: A new metaheuristic algorithm for solving optimization problems. Appl Intell. 2021;51(3):1531–1551.

[203] Zhang L, Wang J, Niu X, Liu Z. Ensemble wind speed forecasting with multi-objective Archimedes optimization algorithm and sub-model selection. Appl Energy. 2021;301(4):117449.

[204] Qais MH, Hasanien HM, Alghuwainem S. Transient search optimization: A new metaheuristic optimization algorithm. Appl Intell. 2020;50:3926–3941.

[205] Qais MH, Hasanien HM, Alghuwainem S. Transient search optimization for electricalparameters estimation of photovoltaic module based on datasheet values. Energy Convers Manag. 2020;214(june):112904.

[206] Eskandar H, Sadollah A, Bahreininejad A, Hamdi M. Water cycle algorithm–A novel metaheuristic optimization method for solving constrained engineering optimization problems. Comput Struct. 2012;110(1):151–166.

[207] Osaba E, Del Ser J, Sadollah A, Bilbao MN, Camacho D. A discrete water cycle algorithm for solving the symmetric and asymmetric traveling salesman problem. Appl Soft Comput. 2018;71(1-2):277–290.

[208] Yg Z, Tang J, Liao R-P, Ziang M-F, Zhang Y, Wang X-M, Su Z-Y. Application of an enhanced BP neural network model with water cycle algorithm on landslide prediction. Stoch Env Res Risk A. 2021;35(61-65):1273–1291.

[209] Simon D. Biogeography-based optimization. IEEE Trans Evol Comput. 2008;12:702–713.

[210] Albashish D, Hammouri AI, Braik M, Atwan J, Sahran S. Binary biogeography-based optimization based SVM-RFE for feature selection. Appl Soft Comput. 2021;101(2):107026.

[211] Ghobaei-Arani M. A workload clustering based resource provisioning mechanism using biogeography based optimization technique in the cloud based systems. Soft Comput. 2021;25(4):3813–3830.

[212] An Y, Chen X, Li Y, Han Y, Zhang J, Shi H. An improved non-dominated sorting biogeography-based optimization algorithm for the (hybrid) multi-objective flexible job-shop scheduling problem. Appl Soft Comput. 2021;99:106869.

[213] Bayraktar Z, Komurcu M, Werner DH. Wind Driven Optimization (WDO): A novel nature-inspired optimization algorithm and its application to electromagnetics. Paper presented at: 2010 IEEE Antennas and Propagation Society International Symposium; 2010; Toronto, ON, Canada.

[214] Bayraktar Z, Komurcu M, Bossard JA, Werner DH. The wind driven optimization technique and its application in electromagnetics. IEEE Trans Antennas Propag. 2013;61(5):2745–2757.

[215] Bhandari AK, Singh VK, Kumar A, Singh GK. Cuckoo search algorithm and wind driven optimization based study of satellite image segmentation for multilevel thresholding using Kapur’s entropy. Expert Syst Appl. 2014;41:3538–3560.

[216] Mirjalili S. SCA: A sine cosine algorithm for solving optimization problems. Knowl-Based Syst. 2016;96:120–133.

[217] Belazzoug M, Touahria M, Nouioua F, Brahimi M. An improved sine cosine algorithm to select features for text categorization. J King Saud Univ-Comput Inform Sci. 2020;32:454–464.

[218] Aydin O, Gozde H, Dursun M, Taplamacioglu MC. Comparative parameter estimation of single diode PV-cell model by using sine-cosine algorithm and whale optimization algorithm. Paper presented at: 2019 6th International Conference on Electrical and Electronics Engineering (ICEEE); 2019; Istanbul, Turkey.

[219] Suid M, Tumari M, Ahmad M. A modified sine cosine algorithm for improving wind plant energy production. Indones J Electrical Eng Comput Sci. 2019;16(1):101–106.

[220] Ewees AA, Abd Elaziz M, Al-Qaness MAA, Khalil HA, Kim S. Improved artificial bee colony using sine-cosine algorithm for multi-level thresholding image segmentation. IEEE Access. 2020;8:26304–26315. 10.1109/ACCESS.2020.3007928 PMC8043509 34192114

[221] Ahmadianfar I, Bozorg-Haddad O, Chu X. Gradient-based optimizer: A new metaheuristic optimization algorithm. Inf Sci. 2020;540:131–159.

[222] Ahmadianfar I, Gong W, Heidari AA, Golilarz NA, Samadi-Koucheksaraee A, Chen H. Gradient-based optimization with ranking mechanisms for parameter identification of photovoltaic systems. Energy Rep. 2021;7(12):3979–3997.

[223] Zhou W, Wang P, Heidari AA, Zhao X, Turabieh H, Chen H. Random learning gradient based optimization for efficient design of photovoltaic models. Energy Convers Manag. 2021;230:113751.

[224] Helmi AM, Al-Qaness MA, Dahou A, Damasevicius R, Krilaviˇcius T, Elaziz MA. A novel hybrid gradient-based optimizer and grey wolf optimizer feature selection method for human activity recognition using smartphone sensors. Entropy. 2021;23(8):1065. 34441205 10.3390/e23081065 PMC8393762

[225] Noble C, Olejarz J, Esvelt KM, Church GM, Nowak MA. Evolutionary dynamics of CRISPR gene drives. Sci Adv. 2017;3(4):e1601964. 28435878 10.1126/sciadv.1601964 PMC5381957

[226] Siddique N, Adeli H. Nature inspired computing: An overview and some future directions. Cogn Comput. 2015;7(6):706–714. 10.1007/s12559-015-9370-8 PMC4675795 26693257

[227] Lieberman E, Hauert C, Nowak MA. Evolutionary dynamics on graphs. Nature. 2005;433(7023):312–316. 15662424 10.1038/nature03204

[228] Harpaz R, Nguyen MN, Bahl A, Engert F. Precise visuomotor transformations underlying collective behavior in larval zebrafish. Nat Commun. 2021;12(1):1–14. 34772934 10.1038/s41467-021-26748-0 PMC8590009

[229] Rojas OL, Pröbstel A-K, Porfilio EA, Wang AA, Charabati M, Sun T, DSW L, Galicia G, Ramaglia V, Ward LA LA. Recirculating intestinal IgA-producing cells regulate neuroinflammation via IL-10. Cell. 2019;176:610–624. 30612739 10.1016/j.cell.2018.11.035 PMC6903689

[230] Kao CY, Los FC, Aroian RV. Nervous about immunity: Neuronal signals control innate immune system. Nat Immunol. 2008;9:1329–1330. 19008931 10.1038/ni1208-1329

[231] Koren T, Amer M, Krot M, Krot M, Boshnak N, Ben-Shaanan TL, Azulay-Debby H, Zalayat I, Avishai E, Hajjo H, et al. Insular cortex neurons encode and retrieve specific immune responses. Cell. 2021;184:5902–5915. 34752731 10.1016/j.cell.2021.10.013

[232] Gütig R. Spiking neurons can discover predictive features by aggregate-label learning. Science. 2016;351:aab4113. 26941324 10.1126/science.aab4113

[233] Qin L, Wang Z, Yan R, Tang H. Attention-based deep spiking neural networks for temporal credit assignment problems. IEEE Trans Neural Netw Learn Syst. 2023. 10.1109/TNNLS.2023.3240176 37022405

[234] Zhu C, Huang W, Li W, Yu X, Li X. Light-emitting artificial synapses for neuromorphic computing. Research. 2022;2022:9786023. 38617552 10.34133/2022/9786023 PMC11014729

[235] Nadji-Tehrani M, Eslami A. A brain-inspired framework for evolutionary artificial general intelligence. IEEE Trans Neural Net Learn Syst. 2020;31:5257–5271. 10.1109/TNNLS.2020.2965567 32175876

[236] Kasabov N, Dhoble K, Nuntalid N, Mohemmed A. Evolving probabilistic spiking neural networks for spatio-temporal pattern recognition: A preliminary study on moving object recognition. In: *International Conference on Neural Information Processing (ICONIP 2011)*. Heidelberg: Springer; 2011. p. 230–239.

[237] Kasabov N. Neucube evospike architecture for spatio-temporal modelling and pattern recognition of brain signals. In: *Artificial Neural Networks in Pattern Recognition: 5th INNS IAPR TC 3 GIRPR Workshop, ANNPR 2012*. Heidelberg: Springer; 2012. p. 225–243.

[238] Song Y, Wu Y, Guo Y, Yan R, Suganthan PN, Zhang Y, Pedrycz W, das S, Mallipeddi R, Ajani OS, et al. Reinforcement learning-assisted evolutionary algorithm: A survey and research opportunities. Swarm Evol Comput. 2024;86:101517.

[239] Fan C, Yao L, Zhang J, Zhen Z, Wu X. Advanced reinforcement learning and its connections with brain neuroscience. Research. 2023;6:0064. 36939448 10.34133/research.0064 PMC10017102

[240] Zanin M, Correia M, Sousa PA, Cruz J. From phenotype to genotype in complex brain networks. Sci Rep. 2016;6:19790. 26795752 10.1038/srep19790 PMC4726251

[241] Davidson EH, Erwin DH. Gene regulatory networks and the evolution of animal body plans. Science. 2006;311(5762):796–800. 16469913 10.1126/science.1113832

[242] Margetts S. *Adaptive genotype to phenotype mappings for evolutionary algorithms.* Cardiff (UK): Cardiff University; 2001.

[243] Bubeck S, Chandrasekaran V, Eldan R, et al. Sparks of artificial general intelligence: Early experiments with gpt-4. arXiv. 2023. https://doi.org/10.48550/arXiv.2303.12712.

[244] Yang C, Wang X, Lu Y, et al. Large language models as optimizers. arXiv. 2023. 10.48550/arXiv.2309.03409

[245] Woeginger GJ. Exact algorithms for NP-hard problems: A survey. In: *Combinatorial optimization—Eureka, you shrink*! Vol. 2570. Heidelberg: Springer; 2003. p. 185–207.

[246] Yang Z, Tang K, Yao X. Multilevel cooperative coevolution for large scale optimization. Paper presented at: 2008 IEEE Congress on Evolutionary Computation (IEEE World Congress on Computational Intelligence); 2008; Hong Kong.

[247] Omidvar MN, Li X, Yang Z, Yao X. Cooperative co-evolution for large scale optimization through more frequent random grouping. Paper presented at: IEEE Congress on Evolutionary Computation; 2010; Barcelona, Spain.

[248] Tian Y, Si L, Zhang X, Cheng R, He C, Tan KC, Jin Y. Evolutionary large-scale multi-objective optimization: A survey. ACM Comput Surv. 2021;54(8):1–34.

[249] Gong YJ, Chen WN, Zhan ZH, Zhang J, Li Y, Zhang Q, Li JJ. Distributed evolutionary algorithms and their models: A survey of the state-of-the-art. Appl Soft Comput.2015;34:286–300.

[250] Hong H and Jiang M. Pre-evolved model for complex multi-objective optimization problems. arXiv. 2023. 10.48550/arXiv.2312.06125

[251] Huang Y, Zhang Z, Jiao A, Ma Y, Cheng R. A comparative visual analytics framework. For evaluating evolutionary processes in multi-objective optimization. IEEE Trans Vis Comput Graph. 2024;30:661–671. 37874721 10.1109/TVCG.2023.3326921

[252] Bian C, Zhou Y, Li M, and Qian C. Stochastic population update can provably be helpful in multi-objective evolutionary algorithms. Paper presented at: Proceedings of the Thirty-Second International Joint Conference on Artificial Intelligence, IJCAI-23; 2023; Macao.

[253] Bali KK, Ong YS, Gupta A, Tan PS. Multifactorial evolutionary algorithm with online transfer parameter estimation: MFEA-II. IEEE Trans Evol Comput. 2019;24(1):69–83.

[254] Wang C, Liu J, Wu K, Wu Z. Solving multitask optimization problems with adaptive knowledge transfer via anomaly detection. IEEE Trans Evol Comput. 2022;26(2):304–318.

[255] Zhao H, Ning X, Liu X, Wang C, Liu J. What makes evolutionary multi-task optimization better: A comprehensive survey. Appl Soft Comput. 2023;145:110545.

[256] Feng L, Gupta A, Tan KC, Ong YS. Ong YS. *Evolutionary multi-task optimization: Foundations and methodologies*. Singapore: Springer; 2023.

[257] Ruder S. An overview of multi-task learning in deep neural networks. arXiv. 2017. 10.48550/arXiv.1706.05098

[258] Sener O, Koltun V. Multi-task learning as multi-objective optimization. *Adv Neural Inf Process Syst*. 2018;31.

[259] Wang C, Jiao L, Zhao J, Li L, Liu X, Liu F, Yang S. Bi-level multiobjective evolutionary learning: A case study on multitask graph neural topology search. IEEE Trans Evol Comput. 2024;28:208–222.

[260] Raghul S, Jeyakumar G. Parallel and distributed computing approaches for evolutionary algorithms—A review. Soft Comput Theor Appl. 2022;1380:433–445.

[261] He X, Zheng Z, Chen C, Zhou Y, Luo C, Lin Q. Distributed evolution strategies for black-box stochastic optimization. IEEE Trans Parallel Distrib Syst. 2022;33:3718–3731.

[262] Santander-Jimenez S, Vega-Rodrıguez MA. Comparative analysis of intra-algorithm parallel multiobjective evolutionary algorithms: Taxonomy implications on bioinformatics scenarios. IEEE Trans Parallel Distrib Syst. 2019;30:63–78.

[263] Zhou Y, Ren Y, Xu M, Feng G. An improved NSGA-III algorithm based on deep Q networks for cloud storage optimization of Blockchain. IEEE Trans Parallel Distrib Syst. 2023;34(5):1406–1419.

[264] Gao J, Huang C, Tang M, Tan SH, Yao X, and Wei X. EFFL: Egalitarian fairness in federated learning for mitigating Matthew effect. arXiv. 2023. 10.48550/arXiv.2309.16338

[265] Liu Q, Yan Y, Ligeti P, Jin Y. A secure federated data-driven evolutionary multi-objective optimization algorithm. IEEE Trans Emerg Topics Comput Intell. 2024;8:191–205.

[266] He X, Zhao K, Chu X. AutoML: A survey of the state-of-the-art. Knowl-Based Syst. 2021;212:106622.

[267] Elsken T, Metzen JH, Hutter F. Neural architecture search: A survey. J Machine Learn Res. 2019;20:1997–2017.

[268] Xu Y, Ma Y. Evolutionary neural architecture search combining multi-branch ConvNet and improved transformer. Sci Rep. 2023;13:15791. 37737271 10.1038/s41598-023-42931-3 PMC10516961

[269] Xue Y, Chen C, Slowik A. Neural architecture search based on a multi-objective evolutionary algorithm with probability stack. IEEE Trans Evol Comput. 2023;27(4):778–786.

[270] Chen A, Dohan D, So D. EvoPrompting: Language models for code-level neural architecture search. In: Oh A, Naumann T, Globerson A, Saenko K, Hardt M, Levine S, editors. *Advances in neural information processing systems*. New York: Curran Associates Inc.; 2023. p. 7787–7817.

[271] Jawahar G, Abdul-Mageed M, Lakshmanan LV, and Ding D. LLM performance predictors are good initializers for architecture search. arXiv. 2023. 10.48550/arXiv.2310.16712

[272] Xu M, Rangamani A, Liao Q, Galanti T, Poggio T. Dynamics in deep classifiers trained with the square loss: Normalization. low rank, neural collapse, and generalization bounds. Research. 2023;6:0024. 37223467 10.34133/research.0024 PMC10202460

[273] Xue K, Qian C, Xu L, and Fei X. Evolutionary gradient descent for non-convex optimization. Paper presented at: Proceedings of the Thirtieth International Joint Conference on Artificial Intelligence, IJCAI-21; 2021; Montreal, Canada.

[274] Bochinski E, Senst T, and Sikora T. Hyper-parameter optimization for convolutional neural network committees based on evolutionary algorithms. Paper presented at: 2017 IEEE International Conference on Image Processing (ICIP); 2017; Beijing, China.

[275] Schranz M, Umlauft M, Sende M, Elmenreich W. Swarm robotic behaviors and current applications. Front Robot AI. 2020;7:36. 33501204 10.3389/frobt.2020.00036 PMC7805972

[276] Pellegrinelli S, Pedrocchi N, Tosatti LM, Fischer A, Tolio T. Multi-robot spot-welding cells for car-body assembly: Design and motion planning. Robot Comput Integr Manuf. 2017;44:97–116.

[277] Wehner M, Truby RL, Fitzgerald DJ, Mosadegh B, Whitesides GM, Lewis JA, Wood RJ. An integrated design and fabrication strategy for entirely soft, autonomous robots. Nature. 2016;536(7617):451–455. 27558065 10.1038/nature19100

[278] Dorigo M, Theraulaz G, Trianni V. Swarm robotics: Past, present, and future [point of view]. Proc IEEE. 2021;109(7):1152–1165.

[279] Cheraghi AR, Shahzad S, and Graffi K. Past, present, and future of swarm robotics. In: *Intelligent Systems and Applications: Proceedings of the 2021 Intelligent Systems Conference (IntelliSys) Volume 3*. Cham: Springer, 2022. p. 190–233.

[280] Bredeche N, Fontbonne N. Social learning in swarm robotics. Philos Trans R Soc B. 2022;377:20200309. 10.1098/rstb.2020.0309 PMC8666954 34894730

[281] Li FF, Du Y, Jia KJ. Path planning and smoothing of mobile robot based on improved artificial fish swarm algorithm. Sci Rep. 2022;12(1):659. 35027589 10.1038/s41598-021-04506-y PMC8758729

[282] Yang GZ, Bellingham J, Dupont PE, Fischer P, Floridi L, Full R, Jacobstein N, Kumar V, McNutt M, Merrifield R, et al. The grand challenges of science robotics. Sci Robot. 2018;3:eaar7650. 33141701 10.1126/scirobotics.aar7650

[283] Zhu M, Biswas S, Dinulescu SI, Kastor N, Hawkes EW, Visell Y. Soft, wearable robotics and haptics: Technologies, trends, and emerging applications. Proc IEEE. 2022;110:246–272.

[284] Yang GZ, Dario P, Kragic D. Social robotics—Trust, learning, and social interaction. Sci Robot. 2018;3:eaau8839. 33141725 10.1126/scirobotics.aau8839

[285] Reina A. Robot teams stay safe with blockchains. Nat Mach Intell. 2020;2:240–241.

[286] Talamali MS, Saha A, Marshall JA, Reina A. When less is more: Robot swarms adapt better to changes with constrained communication. Sci Robot. 2021;6:eabf1416. 34321345 10.1126/scirobotics.abf1416

[287] Yan X, Ma J, Wu T, Zhang A, Wu J, Chin M, Zhang Z, Dubey M, Wu W, Chen MSW, et al. Reconfigurable stochastic neurons based on tin oxide/MoS2 hetero memristors for simulated annealing and the Boltzmann machine. Nat Commun. 2021;12(1):5710. 34588444 10.1038/s41467-021-26012-5 PMC8481256

[288] Haouari M, Mhiri M. A particle swarm optimization approach for predicting the number of COVID-19 deaths. Sci Rep. 2021;11(1):1–13. 34400735 10.1038/s41598-021-96057-5 PMC8367975

[289] Zhou H, Yang W, Sun L, Jing X, Li G, Cao L. Reliability optimization of process parameters for marine diesel engine block hole system machining using improved PSO. Sci Rep. 2021;11(1):1–13. 34754070 10.1038/s41598-021-01567-x PMC8578651

[290] Liu B, Wang L, Jin YH. An effective PSO-based memetic algorithm for flow shop scheduling. IEEE Trans Syst Man Cybern B Cybern. 2007;37(1):18–27. 17278555 10.1109/tsmcb.2006.883272

[291] Dagal I, Akın B, Akboy E. MPPT mechanism based on novel hybrid particle swarm optimization and salp swarm optimization algorithm for battery charging through simulink. Sci Rep. 2022;12(1):1–17. 35177713 10.1038/s41598-022-06609-6 PMC8854737

[292] Merkle D, Middendorf M, Schmeck H. Ant colony optimization for resource-constrained project scheduling. IEEE Trans Evol Comput. 2002;6:333–346.

[293] Aguilar J, Monaenkova D, Linevich V, Savoie W, Dutta B, Kuan HS, Betterton MD, Goodisman MAD, Goldman DI. Collective clog control: Optimizing traffic flow in confined biological and robophysical excavation. Science. 2018;361(6403):672–677. 30115804 10.1126/science.aan3891

[294] Rathee M, Kumar S, Gandomi AH, Dilip K, Balusamy B, Patan R. Ant colony optimization based quality of service aware energy balancing secure routing algorithm for wireless sensor networks. IEEE Trans Eng Manag. 2019;68:170–182.

[295] Baykasoglu A, Ozbakir L, Tapkan P. Artificial bee colony algorithm and its application to generalized assignment problem. In: Chan FT, Tiwari MK, editorsSwarm intelligence Rijeka: IntechOpen; 2007.

[296] Alrosan A, Alomoush W, Norwawi N, Alswaitti M, Makhadmeh SN. An improved artificial bee colony algorithm based on mean best-guided approach for continuous optimization problems and real brain MRI images segmentation. Neural Comput Appl. 2021;33:1671–1697.

[297] Gao W, Sheng H, Wang J, Wang S. Artificial bee colony algorithm based on novel mechanism for fuzzy portfolio selection. IEEE Trans Fuzzy Syst. 2018;27:966–978.

[298] Al-Tashi Q, Kadir SJA, Rais HM, Mirjalili S, Alhussian H. Binary optimization using hybrid grey wolf optimization for feature selection. IEEE Access. 2019;7:39496–39508.

[299] Hosseini-Hemati S, Sheisi GH, Karimi S. Allocation-based optimal reactive power dispatch considering polynomial load model using improved grey wolf optimizer. Iran J Sci Technol Trans Electr Eng. 2021;45:921–944.

[300] Bacanin N, Bezdan T, Tuba E, Strumberger I, Tuba M,Zivkovic M. Task Scheduling in cloud computing environment by grey wolf optimizer. Paper presented at: 2019 27th Telecommunications Forum (TELFOR); 2019; Belgrade, Serbia.

[301] Ewees AA, Sahlol AT, Amasha MA. A bio-inspired moth-flame optimization algorithm for arabic handwritten letter recognition. Paper presented at: 2017 International Conference on Control, Artificial Intelligence, Robotics & Optimization (ICCAIRO); 2017; Prague, Czech Republic.

[302] Parmar SA, Pandya MH, Bhoye M, Trivedi IN, Jangir P, Ladumor D. Optimal active and reactive power dispatch problem solution using Moth-Flame Optimizer algorithm. Paper presented at: 2016 International Conference on Energy Efficient Technologies for Sustainability (ICEETS); 2016; Nagercoil, India.

[303] Yıldız BS, Yıldız AR. Moth-flame optimization algorithm to determine optimal machining parameters in manufacturing processes. Mater Test. 2017;59:425–429.

[304] Raghavan S, Sarwesh P, Marimuthu C, Chandrasekaran K. Bat algorithm for scheduling workflow applications in cloud. Paper presented at: 2015 International Conference on Electronic Design, Computer Networks & Automated Verification (EDCAV); 2015; Shillong, India.

[305] Tuba E, Tuba M, Simian D. Adjusted bat algorithm for tuning of support vector machine parameters. Paper presented at: 2016 IEEE Congress on Evolutionary Computation (CEC); 2016; Vancouver, BC, Canada.

[306] Nakamura RYM, Pereira LAM, Costa KA, Rodrigues D, Papa JP, Yang XS. BBA: A binary bat algorithm for feature selection. Paper presented at: 2012 25th SIBGRAPI Conference on Graphics, Patterns and Images; 2012; Ouro Preto, Brazil.

[307] Yang XS. Bat algorithm for multi-objective optimisation. Int J Bio-Insp Comput. 2011;3(5):267–274.

[308] Jia H, Xing Z, Song W. A new hybrid seagull optimization algorithm for feature selection. IEEE Access. 2019;7:49614–49631.

[309] Panagant N, Pholdee N, Bureerat S, Kaen K, Yıldız AR, Sait SM. Seagull optimization algorithm for solving real-world design optimization problems. Mater Test. 2020;62(6):640–644.

[310] Dhiman G, Singh KK, Soni M, Nagar A, Dehghani M, Slowik A, Kaur A, Sharma A, Houssein EH, Cengiz K. MOSOA: A new multi-objective seagull optimization algorithm. Expert Syst Appl. 2021;167:114150.

[311] Cao Y, Li Y, Zhang G, Jermsittiparsert K, Razmjooy N. Experimental modeling of PEM fuel cells using a new improved seagull optimization algorithm. Energy Rep. 2019;5:1616–1625.

[312] Strumberger I, Tuba E, Bacanin N, Beko M, Tuba M. Monarch butterfly optimization algorithm for localization in wireless sensor networks. Paper presented at: 2018 28th International Conference Radioelektronika (RADIOELEKTRONIKA); 2018; Prague, Czech Republic.

[313] Strumberger I, Tuba M, Bacanin N, Tuba E. Cloudlet scheduling by hybridized monarch butterfly optimization algorithm. J Sens Actuator Netw. 2019;8(3):44.

[314] Ehteram M, Karami H, Mousavi SF, Farzin S, Kisi O. Optimization of energy management and conversion in the multi-reservoir systems based on evolutionary algorithms. J Clean Prod. 2017;168:1132–1142.

[315] Duan H, Wang X. Echo state networks with orthogonal pigeon-inspired optimization for image restoration. IEEE Trans Neur Netw Learn Syst. 2015;27(11):2413–2425. 10.1109/TNNLS.2015.2479117 26529785

[316] Qiu H, Duan H. A multi-objective pigeon-inspired optimization approach to UAV distributed flocking among obstacles. Inf Sci. 2020;509:515–529.

[317] Cui Z, Zhang J, Wang Y, Cao Y, Cai X, Zhang W, Chen J. A pigeon-inspired optimization algorithm for many-objective optimization problems. Sci China Inf Sci. 2019;62(7):1–3.

[318] Deng Y, Duan H. Control parameter design for automatic carrier landing system via pigeon-inspired optimization. Nonlin Dyn. 2016;85(1):97–106.

[319] Chintam JR, Daniel M. Real-power rescheduling of generators for congestion management using a novel satin bowerbird optimization algorithm. Energies. 2018;11(1):183.

[320] Thakkar N, Paliwal P. Application of Satin Bowerbird Algorithm for Optimal Sizing of a Solar-Biomass based Microgrid. Paper presented at: 2021 13th IEEE PES Asia Pacific Power & Energy Engineering Conference (APPEEC); 2021; Thiruvananthapuram, India.

[321] Moosavi SKR, Zafar MH, Akhter MN, Hadi SF, Khan NM, Sanfilippo F. A novel artificial neural network (ANN) using the mayfly algorithm for classification. Paper presented at: 2021 International Conference on Digital Futures and Transformative Technologies (ICoDT2); 2021; Islamabad, Pakistan.

[322] Shaheen MA, Hasanien HM, El Moursi M, El-Fergany AA. Precise modeling of PEM fuel cell using improved chaotic MayFly optimization algorithm. Int J Energy Res. 2021;45(11):18754–18769.

[323] Houssein EH, Helmy BE, Oliva D, Elngar AA, Shaban H. A novel black widow optimization algorithm for multilevel thresholding image segmentation. Expert Syst Appl. 2021;167:114159.

[324] Ravikumar S, Kavitha D. IOT based autonomous car driver scheme based on ANFIS and black widow optimization. J Ambient Intell Humaniz Comput. 2021;1–14.

[325] Diab AAZ, Sultan HM, Do TD, Kamel OM, Mossa MA. Coyote optimization algorithm for parameters estimation of various models of solar cells and PV modules. IEEE Access. 2020;8:111102–111140.

[326] RCT S, Macedo CA, Santos Coelho L, Pierezan J, Mariani VC. Binary coyote optimization algorithm for feature selection. Pattern Recogn. 2020;107:107470.

[327] Gu¨ven¸c U, Kaymaz E. Economic dispatch integrated wind power using coyote optimization algorithm. Paper presented: 2019 7th International Istanbul Smart Grids and Cities Congress and Fair (ICSG); 2019; Istanbul, Turkey.

[328] Tuba E, Stanimirovic Z. Elephant herding optimization algorithm for support vector machine parameters tuning. Paper presented at: 2017 9th International Conference on Electronics, Computers and Artificial Intelligence (ECAI); 2017; Targoviste, Romania.

[329] Correia SD, Beko M, Silva Cruz LA, Tomic S. Elephant herding optimization for energy-based localization. Sensors. 2018;18:2849. 30158442 10.3390/s18092849 PMC6163308

[330] Meena NK, Parashar S, Swarnkar A, Gupta N, Niazi KR. Improved elephant herding optimization for multiobjective DER accommodation in distribution systems. IEEE Trans Industr Inform. 2017;14(3):1029–1039.

[331] Santosa B, Ningrum MK. Cat swarm optimization for clustering. Paper presented at: 2009 International Conference of Soft Computing and Pattern Recognition; 2009 Dec 4–7; Malacca, Malaysia.

[332] Bilgaiyan S, Sagnika S, Das M. Workflow scheduling in cloud computing environment using cat swarm optimization. Paper presented at: 2014 IEEE International Advance Computing Conference (IACC); 2014; Gurgaon, India.

[333] Guo L, Meng Z, Sun Y, Wang L. Parameter identification and sensitivity analysis of solar cell models with cat swarm optimization algorithm. Energy Convers Manag. 2016;108(3):520–528.

[334] Marichelvam MK, Prabaharan T, Yang XS. A discrete firefly algorithm for the multi objective hybrid flowshop scheduling problems. IEEE Trans Evol Comput. 2013;18(2):301–305.

[335] Sundararaj V et al. An efficient threshold prediction scheme for wavelet based ECG signal noise reduction using variable step size firefly algorithm. Int J Intell Eng Syst.2016;9(3):117–126.

[336] Vincentius R, Nugraha A, Talitha P, Margo P, Ardyono P, Mauridhi H. Recognition of electric machines boundary as the constraint of over current relay coordination in real industrial application with serial firefly algorithm optimization. Paper presented at: 2019 IEEE 12th International Symposium on Diagnostics for Electrical Machines, Power Electronics and Drives (SDEMPED); 2019; Toulouse, France.

[337] Tasnim M, Rouf S, Rahman MS. A CLONALG-based approach for the set covering problem. Paper presented at: 2012 15th International Conference on Computer and Information Technology (ICCIT); 2012; Chittagong, Bangladesh.

[338] Perez-Caceres L, Riff MC. Solving scheduling tournament problems using a new version of CLONALG. Connect Sci. 2015;27(1):5–21.

[339] Zhang L, Zhong Y, Huang B, Li P. A resource limited artificial immune system algorithm for supervised classification of multi/hyper-spectral remote sensing imagery. Int J Remote Sens. 2007;28(7):1665–1686.

[340] Kanwal S, Khan F, Alamri S, Dashtipur K, Gogate M. COVID-opt-aiNet: A clinical decision support system for COVID-19 detection. Int J Imaging Syst Technol. 2022;32(18):444–461. 35465215 10.1002/ima.22695 PMC9015255

[341] Selahshoor F, Jazayeriy H, Omranpour H. Intrusion detection systems using real-valued negative selection algorithm with optimized detectors. Paper presented at: 2019 5th Iranian Conference on Signal Processing and Intelligent Systems (ICSPIS); 2019; Shahrood, Iran.

[342] Gao XZ, Wang X, Ovaska SJ, Arkkio A, Zenger K, Wang X. A Negative Selection Algorithm-based motor fault detection scheme. Paper presented at: 2011 Seventh International Conference on Natural Computation; 2011; Shanghai, China.

[343] Greensmith J, Aickelin U, Tedesco G. Information fusion for anomaly detection with the dendritic cell algorithm. Inform Fusion. 2010;11(1):21–34.

[344] Mokhtar M, Bi R, Timmis J, Tyrrell AM. A modified dendritic cell algorithm for online error detection in robotic systems. Paper presented at: 2009 IEEE Congress on Evolutionary Computation; 2009; Trondheim, Norway. 10.1109/CEC.2009.4983194

[345] Cheng Z, Sun Y, Liu Y. Path planning based on immune genetic algorithm for UAV. Paper presented at: 2011 International Conference on Electric Information and Control Engineering; 2011; Wuhan.

[346] Chen F, Tang B, Chen R. A novel fault diagnosis model for gearbox based on wavelet support vector machine with immune genetic algorithm. Measurement. 2013;46(1):220–232.

[347] Jadhav HT, Sharma U, Patel J, Roy R. Brain storm optimization algorithm based economic dispatch considering wind power. Paper presented at: 2012 IEEE International Conference on Power and Energy (PECon); 2012; Kota Kinabalu, Malaysia.

[348] Sun C, Duan H, Shi Y. Optimal satellite formation reconfiguration based on closed-loop brain storm optimization. IEEE Comput Intell Mag. 2013;8(8):39–51.

[349] Arpaia P, Lucariello G, Zanesco A. Automatic fault isolation by cultural algorithms with differential influence. IEEE Trans Instrum Meas. 2007;56(5):1573–1582.

[350] Wang W, Song Y, Xue Y, Jin H, Hou J, Zhao M. An optimal vibration control strategy for a vehicle’s active suspension based on improved cultural algorithm. Appl Soft Comput. 2015;28:167–174.

[351] Oloruntoba O, Cosma G, Liotta A. Clan-based cultural algorithm for feature selection. Paper presented at: 2019 International Conference on Data Mining Workshops (ICDMW); 2019; Beijing, China.

[352] Maheri MR, Talezadeh M. An enhanced imperialist competitive algorithm for optimum design of skeletal structures. Swarm Evol Comput. 2018;40(4598):24–36.

[353] Shao W, Pi D, Shao Z. A hybrid discrete optimization algorithm based on teaching–probabilistic learning mechanism for no-wait flow shop scheduling. Knowl-Based Syst. 2016;107:219–234.

[354] Han KH, Kim JH. Quantum-inspired evolutionary algorithm for a class of combinatorial optimization. IEEE Trans Evol Comput. 2002;6(6):580–593.

[355] Arya A, Botelho L, Canete F, Kapadia D, Salehi O. Music composition using quantum annealing. arXiv. 2022. 10.48550/arXiv.2201.10557

[356] Singh AK, Saxena D, Kumar J, Gupta V. A quantum approach towards the adap1720 tive prediction of cloud workloads. IEEE Trans Parallel Distrib Syst. 2021;32:2893–2905.

[357] Acharya S, Ganesan S, Kumar DV, Subramanian S. A multi-objective multi-verse optimization algorithm for dynamic load dispatch problems. Knowl-Based Syst. 2021;231(4):107411.

[358] Shareef H, Mutlag AH, Mohamed A. A novel approach for fuzzy logic PV inverter controller optimization using lightning search algorithm. Neurocomputing. 2015;168:435–453.

[359] Hannan MA, Ali JA, Mohamed A, Amirulddin UAU, Tan NML, Uddin MN. Quantum behaved lightning search algorithm to improve indirect field-oriented Fuzzy-PI control for IM drive. IEEE Trans Ind Appl. 2018;54(4):3793–3805.

[360] Moreno SR, Pierezan J, dos Santos Coelho L, Mariani VC. Multi-objective lightning search algorithm applied to wind farm layout optimization. Energy 2021;216:119214.

